# Nanoscale hetero-interfaces for electrocatalytic and photocatalytic water splitting

**DOI:** 10.1080/14686996.2022.2125827

**Published:** 2022-10-04

**Authors:** Baopeng Yang, Dingzhong Luo, Shimiao Wu, Ning Zhang, Jinhua Ye

**Affiliations:** a School of Materials Science and Engineering, Central South University, Changsha, Hunan, P. R. China; b School of Physics and Electronics, Central South University, Changsha, Hunan, P. R. China; c College of Chemistry and Chemical Engineering, Xiamen University, Xiamen, China; d International Center for Materials Nanoarchitectonics (WPI-MANA), National Institute for Materials Science (NIMS), Tsukuba, Ibaraki, Japan

**Keywords:** Hetero-Interface, photocatalysis, electrocatalysis, water splitting, hydrogen energy

## Abstract

As green and sustainable methods to produce hydrogen energy, photocatalytic and electrochemical water splitting have been widely studied. In order to find efficient photocatalysts and electrocatalysts, materials with various composition, size, and surface/interface are investigated. In recent years, constructing suitable nanoscale hetero-interfaces can not only overcome the disadvantages of the single-phase material, but also possibly provide new functionalities. In this review, we systematically introduce the fundamental understanding and experimental progress in nanoscale hetero-interface engineering to design and fabricate photocatalytic and electrocatalytic materials for water splitting. The basic principles of photo-/electro-catalytic water splitting and the fundamentals of nanoscale hetero-interfaces are briefly introduced. The intrinsic behaviors of nanoscale hetero-interfaces on electrocatalysts and photocatalysts are summarized, which are the electronic structure modulation, space charge separation, charge/electron/mass transfer, support effect, defect effect, and synergistic effect. By highlighting the main characteristics of hetero-interfaces, the main roles of hetero-interfaces for electrocatalytic and photocatalytic water splitting are discussed, including excellent electronic structure, efficient charge separation, lower reaction energy barriers, faster charge/electron/mass transfer, more active sites, higher conductivity, and higher stability on hetero-interfaces. Following above analysis, the developments of electrocatalysts and photocatalysts with hetero-structures are systematically reviewed.

## Graphical abstract

**Figure:**
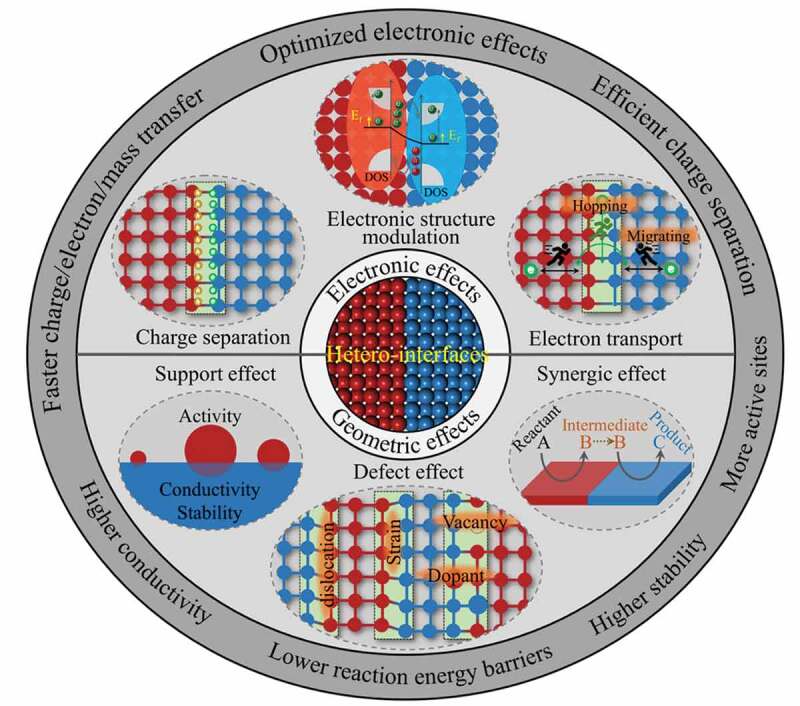

## 1. Introduction

The rapid growing consumption of the fossil fuels causes serious energy crisis and environmental pollution. Green and sustainable energy is important for maintaining clean and livable environments in modern society [1–3]. Molecular hydrogen (H_2_) is deemed to be an ideal sustainable energy due to the feature of carbon-free and high energy density, it will revolutionize the energy structure of the world in the future [4,5]. H_2_ can be directly used as a fuel gas to replace fossil fuels, and it can also be used to store additional energy through a method that integrates variable renewable power into the energy grid [6,7]. Therefore, establishing a technique for H_2_ generation is critical and essential. H_2_ can be created in general from (gasified) coal, natural gas, biomass, or water. At present, the majority of hydrogen is produced by reforming natural gas with steam, this process will inevitably produce harmful byproducts such as carbon dioxide [2,6]. Even though biomass is a renewable resource and can be converted into a wide variety of liquid fuels, it is not capable of meeting the demand for hydrogen on a large scale. Therefore, effective and environmentally friendly methods of obtaining hydrogen should be developed. In principle, H_2_ can be produced by splitting water molecules, this is a completely green and sustainable way to generate hydrogen energy. H_2_O molecules can be split by light and electricity [8,9]. Photocatalytic and electrocatalytic water splitting techniques, which have the benefits of an unlimited water supply, high purity of hydrogen synthesis, and mass production practicability, are efficient and environmentally benign hydrogen production processes [10–12]. Nevertheless, the output of H_2_ from photochemical and electrochemical splitting of water accounts for less than 5% of the total hydrogen supply nowadays [6,13–15]. Therefore, enabling water splitting in the direction of broad industrialisation is a tough problem. The process of water splitting can be broken down into two distinct reactions: the oxygen evolution reaction (OER) and the hydrogen evolution reaction (HER). Because water is very thermodynamically stable, splitting it must overcome an internal energy barrier of 237.2 kJ mol^−1^ [16–18]. Therefore, exploiting catalysts with high activity, low energy cost, and earth abundance is import for the widespread industrialization application of photo- and electrochemical water splitting technology.

**Figure 1.**
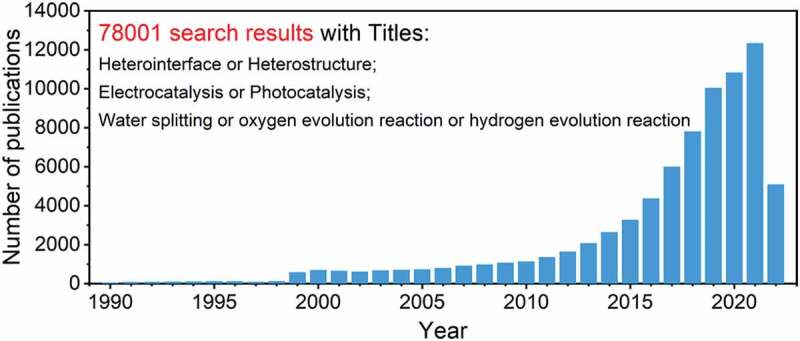
The number of publications searched by the words (Heterointerface or Heterostructure) and (Electrocatalysis or Photocatalysis) and (Water splitting or Oxygen evolution reaction or Hydrogen evolution reaction) in their titles. The data were collected from Web of Science Core Collection on June 28, 2022.

The developed of catalysts for H_2_O splitting should satisfy the photo- and electron- catalytic behaviors thermodynamically and dynamically. At present, TiO_2_, SrTiO_3_, Zn_2_GeO_4_, ZnGa_2_O_4_, WO_3_, CdS and so on have proper energy band structure, which are thermodynamically suitable for the photocatalytic occurrence of water splitting [12,19–23]. For electrolytic materials, although the noble metals-based catalysts such as Pt, Pd, Ru, and Rh are ideal HER or OER electrocatalysts with low overpotential and high efficiency, the high cost and low abundance in earth of noble metals-based materials hinder the widespread utilization in commercial society [6,24,25]. In order to explore catalysts for water splitting with high activity and low cost, the transition metal-based oxides, hydroxides, carbides, phosphate, nitride, and sulfides are extensively studied [26–34]. However, these individual catalysts are still far from meeting the industrial demand. Recently, the fabrication of hetero-interface catalysts by combining several active components has received a lot of attention for water splitting [35–37]. Hetero-structures may dramatically alter the inherent catalytic activities of the individual composition to display distinct physicochemical features toward certain processes, which is regarded as a critical surface engineering method for advancing catalysts with specified functionalities [38–43]. In general, a heterostructure is a structure having a heterointerface or heterojunction generated by stacking or sewing various components together. The distinct properties of heterostructure materials result from the total of the functions given by the individual components as well as the interfacial interaction between the compositions [44]. The unique features of heterostructure catalysts may result in improved or entirely novel water splitting catalytic capabilities. Figure 1 shows the number of publications in the fields of hetero-interfaces for photocatalytic and electrocatalytic water splitting in recent 30 years. The related research began in the 1990s and increased dramatically in 1999. In the following ten years, it maintained an annual publication volume of 1000 to 2000 articles. After entering the 21st century, the number of published papers began to sharply increase year by year and exceeded 10,000 articles in 2019, and in recent years, the number has increased by more than 10,000 per year. So far, a total of nearly 78,000 related papers have been published, indicating that the hetero-interfaces for photocatalytic and electrocatalytic water splitting are attracting interest. Although hetero-interfaces have been widely studied [32,45–53], a systematic summary and understanding of the intrinsic enhancement effects of hetero-interfaces in the field of water splitting was still limited.

In this paper, we review recent reports on the concept, characteristics, and progress of hetero-interfaces in photocatalytic and electrocatalytic water splitting. Firstly, the basic principles of photocatalytic and electrocatalytic water splitting as well as the key factors to determine the catalytic activity is introduced. Then, we summarized the main characteristics of hetero-interfaces, according to their geometry and electronic structure and discussed the key roles of hetero-interface for electrocatalytic and photocatalytic water splitting. Subsequently, we reviewed the recent progress on developing hetero-interface catalysts to realize electrocatalytic and photocatalytic water splitting, highlighting the main characteristics and key roles of hetero-interfaces. Finally, we present some insights for the future development of hetero-interfaces in photo- and electro-catalytic water splitting.

## 2. Fundamentals of nanoscale hetero-interfaces in photo- and electro-catalytic water splitting

Among numerous photo- and electro-catalysts, the composite materials with nanoscale hetero-interfaces have attracted the interest of researchers due to the unique electronic and geometric structure at the interface that are beneficial to facilitate catalytic reactions [36,40,42]. In-depth understanding of the catalytic mechanism of water splitting and the roles of the hetero-interface in water splitting is of great significance for the design of efficient hetero-interfaces catalysts. In this section, the mechanisms, key features, and main functions of nanoscale hetero-interfaces in photo- and electro-catalytic water splitting will be discussed.

### 2.1 Photo- and electro-catalytic water splitting

From the view of thermodynamical reaction, the decomposition of water to produce H_2_ and O_2_ can be described as:

(1) $$H_{2}O\to H_{2}+1\;/\;2O_{2},\;\;\Delta G_{\left(298\;K\right)}^{0}=+273\;kj\;mol^{-1}$$

This overall water splitting reaction can be broken down into two half reactions: the reduction reaction (HER, hydrogen generation) and the oxidation reaction (OER, oxygen generation). Their equations are as follows:

(2) $$2H^{+}+2e^{-}\to H_{2}\left(Reduction\;reaction,HER\right)$$

(3) $$4OH^{-}+4e^{-}\to O_{2}+2H_{2}O\left(Oxidation\;reaction,OER\right)$$

Obviously, this is a thermodynamically unfavorable process accompanied by increased Gibbs free energy, which cannot occur spontaneously and requires extra energy to drive it. In addition, water splitting is a multistep reaction process with high reaction barriers, which reduces the efficiency of water splitting [10,13,53–55]. Therefore, achieving high-efficiency photo- and electro-catalytic water splitting to produce H_2_ energy is considered to be a challenging subject.

**Figure 2.**
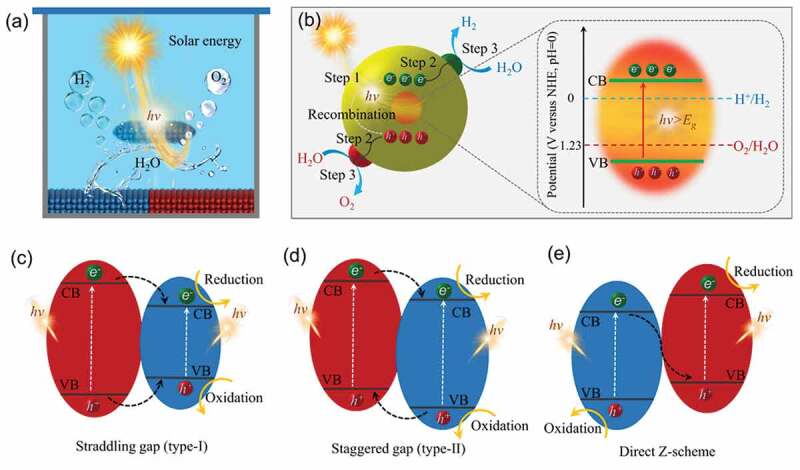
(a) Schematic diagram of photocatalytic water splitting. (b) the main steps and the process of photocatalytic water splitting on catalysts surface. (c-e) the general types of heterojunctions and the electron – hole pairs migration process.

Photocatalytic overall water splitting, like photosynthesis in green plants, is a solar-to-chemical energy conversion process (Figure 2(a)). As illustrated in Figure 2(b), the photocatalytic overall water splitting mechanism consists of three primary steps: (i) light harvesting, (ii) charge separation and migration, and (iii) surface redox reaction [56–58]. Step one, when the light with a certain wavelength is absorbed by catalysts, the photons will interact with the catalysts. When the energy of these adsorbed photons equals or exceeds the bandgap of catalysts, the electrons in the valence band of catalysts would be shifted into the conduction band due to the excitation of photons, thereby generating excited electron-hole pairs. Step two, the generated electrons and holes are separated and gravitated rapidly to the surface-active sites. Step three, these photoexcited carriers and electrons participate in the redox reaction on surface catalytic active sites. To achieve successful photocatalytic water splitting reaction, the catalysts that used for photocatalysis must meet certain requirements, such as visible light absorption capacity, abundant surface active sites, and charge separation efficiency [17,45,56]. Only when photons with energy greater than the band gap are absorbed by the photocatalyst, electrons and holes diffuse to the catalysts surface and induce the water splitting reaction, respectively. In addition, due to the constraints imposed by thermodynamics, a semiconductor photocatalyst that is capable of initiating the water splitting reaction must have a conduction band minimum (CBM) with potential energy lower than that of the H+/H_2_ reduction potential (0 V versus reversible hydrogen (vs. RHE)) and a valence band maximum (VBM) with potential energy higher than that of the O_2_/H_2_O oxidation potential (1.23 V vs. RHE) [10,15,59]. Water splitting performance has been demonstrated to be high when heterojunction photocatalysts comprise multiple semiconductors. A heterojunction structure offers the advantage of preferential band alignment for photocatalytic water splitting in visible light [56]. A major challenge of photocatalysis is reducing the reaction rate during charge separation and migration by recombining the photoexcited electrons in the conduction band (CB) with the holes in the valence band (VB) [52]. A heterojunction can be classified into three types based on the CB and VB levels of two semiconductors. In the type-II heterojunction shown in Figure 2(d), electrons can be transferred from CB of semiconductor A to semiconductor B, while holes can be transferred from the VB of semiconductor B to semiconductor A. It is thus possible to separate electron hole pairs spatially. The electron-hole pairs cannot be separated as efficiently in type-I heterojunctions because both electrons and holes accumulate on the same semiconductor (Figure 2(c)). There is no obvious difference in the band structure between direct Z-schemes and type II heterojunctions; however, charge transfer is in the opposite direction (Figure 2(e)). Z-scheme heterojunctions can both improve charge separation efficiency and retain strong redox ability [58]. Consequently, heterojunction photocatalysts comprised of multiple semiconductors are considered effective for separating and migrating charges. Furthermore, the catalytic ability and amount of active sites on the surface are critical in the overall photocatalytic process, since a lack of active sites might cause electrons and holes to recombine. A rationally constructed hetero-interface between semiconductors can not only improve the intrinsic activity of catalysts by optimizing their electronic structure, but also increase the number of active sites, so that a photocatalytic water splitter can perform better.

Because solar energy is an endless renewable energy source, photocatalytic water splitting is the most optimal way for producing H_2_. However, the low utilization of light energy during photocatalytic water splitting induces a low conversion efficiency and limits its large-scale development. In contrast to photocatalysis, electrocatalytic water splitting is a process that converts electrical energy directly into chemical energy, resulting in better conversion efficiency and higher-purity H_2_. Furthermore, electrical energy may be created affordably from renewable energy sources such as wind, solar, and tidal energy [60,61]. As a result, electrocatalytic water splitting is a more viable technique for converting renewable energy into hydrogen energy, and it has attracted the interest of researchers.

**Figure 3.**
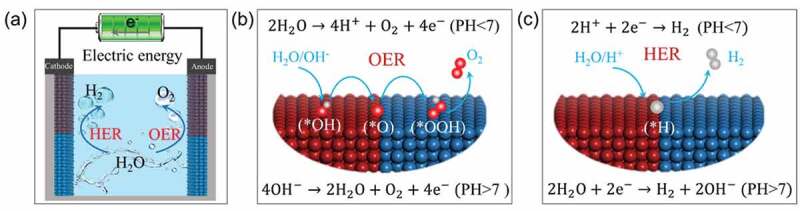
**Electrocatalytic water splitting**. (a) Schematic diagram of electrocatalytic water splitting. (b) OER process in alkaline and acidic solutions. (c) HER process in alkaline and acidic solutions.

Electrochemical water splitting comprises two related half-reactions: HER in the cathode and OER in the anode (Figure 3(a)). These reactions differ depending on the pH value of the electrolytes [16,19]. As shown in Figures 3(b) and 3(c), the water splitting process has many expressions depending on the pH of the electrolytes. In acidic solutions, the HER and OER processes can be expressed as follows:

(4) $$HER:2H^{+}+2e^{-}\to H_{2}(E=0V\;vs.\;RHE)$$

(5) $$OER:2H_{2}O\to 4H^{+}+O_{2}+4e^{-}(E=1.23V\;vs.\;RHE)$$

In neutral or alkaline solutions, the HER and OER processes can be expressed as follows:

(6) $$HER:2H_{2}O+2e^{-}\to H_{2}+2OH^{-}(E=0V\;vs.\;RHE)$$

(7) $$OER:4OH^{-}\to 2H_{2}O+O_{2}+4e^{-}(E=1.23V\;vs.\;RHE)$$

Obviously, HER process needs two electrons participation. The OER process necessitates the transfer of four electrons, which includes the breakdown of the O-H bond and the development of the O-O bond [62–64]. As a result, OER is a thermodynamically unfavorable process that necessitates a high potential in order to overcome the kinetic energy barrier, which is the rate-determining step in total water splitting [65–67]. In general, there are two methods for enhancing electrocatalyst activity: raising the intrinsic activity of each active site and increasing the number of active sites. The intrinsic activity is often related to the electronic structure, charge density, and charge/electron/mass transfer capability of catalysts [11,16,54]. The electronic structure and charge density of materials can be improved through regulating the composition, valence states, bond energy, and defect concentration [47,68–71] The electron/mass transfer capability of catalysts can be enhanced by coupling with some functional materials, such as graphene and some conductive substrates [72,73]. More active sites may enhance the reaction efficiency, the number of active sites is proportional to the active area of electrode, which can be increased by constructing electrodes with large surface area and special morphologies [74–77].

### 2.2 Fundamentals of nanoscale hetero-interfaces

**Figure 4.**
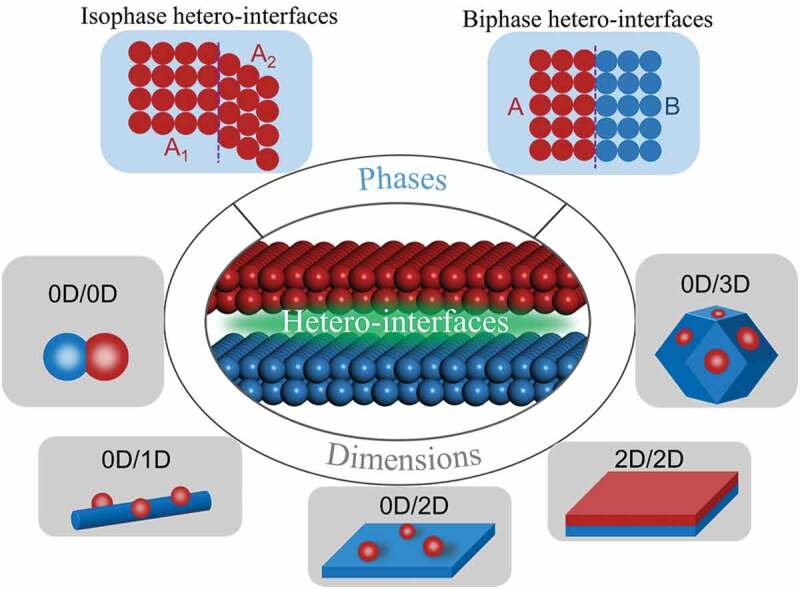
The types of hetero-interfaces from the view of phases and dimensions.

The term “hetero-interfaces” derives from the term “heterojunction” in the field of semiconductor physics, which defines the contact situation between two different materials [39]. The concept of heterostructure has expanded beyond semiconductor physics with the interconnection and merging of knowledge networks. In general, when two dissimilar materials (including semiconductors, conductors as well as insulators) come into contact, a hetero-interface will be formed at the contact interface [78–81]. Hetero-interfaces can be divided into various categories depending on how the interface formed, as shown in Figure 4. According to the types of phases that form the hetero-interface, the hetero-interface can be divided into an isomeric interface and a heterogeneous interface. The isomeric interface is composed of different crystal phase of the same chemical composition. In this case, the materials that make up the hetero-interface have the same chemical composition, but their crystalline forms or crystallinity are different. The heterogeneous interface is composed of two or more different substances. In this case, the chemical composition of materials that make up the hetero-interface are different, the crystal structures of them can be same or different. Moreover, based on the dimension of hetero-interface formation, nanoscale hetero-interfaces can be divided into 0D-0D, 0D-1D, 0D-2D, 0D-3D, and 2D-2D hetero-interfaces. The compositional and structural diversity of the hetero-interface provides more degrees of freedom for catalyst design and improvement.

**Figure 5.**
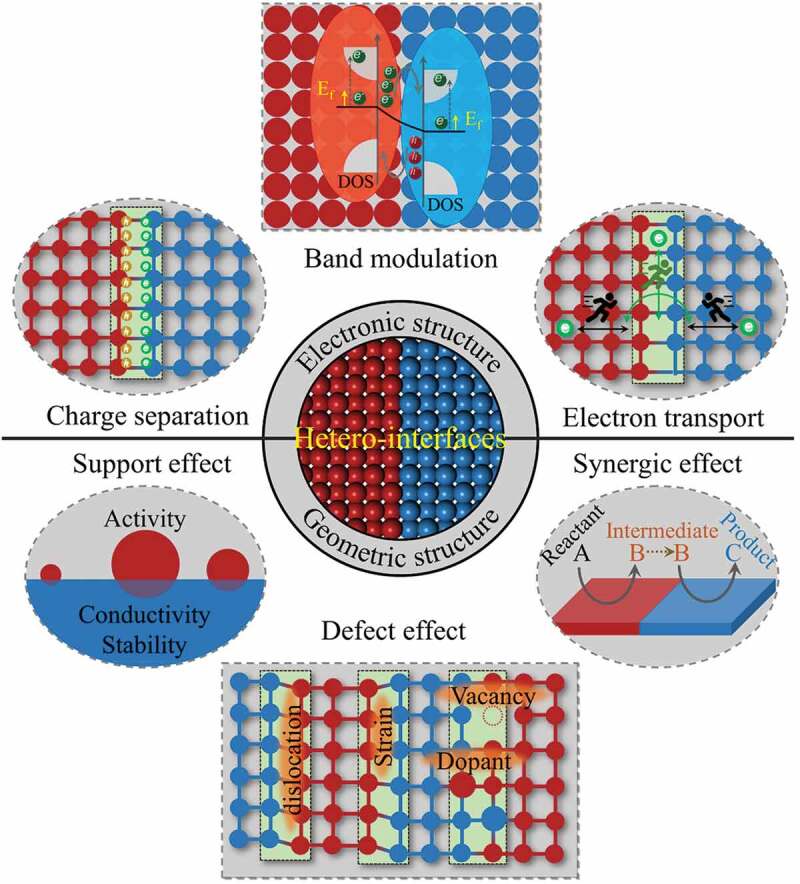
The characteristics of nanoscale hetero-interfaces.

With the development of synthesis methods, various types of hetero-interface catalysts have been successfully designed and synthesized to optimize the catalytic reaction pathways to enhance catalytic performance [35–37,79,80]. Although hetero-interfaces have been shown to have a very significant influences on photo/electrocatalytic properties, the understanding of the intrinsic nature of these enhancement effects was still obscure in the past. Some deeper understandings on the intrinsic characteristics of hetero-interfaces have been revealed with the progress of characterization methods and theoretical investigations, which bring the hetero-interface engineering to a new frontier and hot spot in field of catalysis. Due to the unequal components’ electronic and crystal structures, some special physicochemical features would be generated at the hetero-interfaces [81–86]. According to the electronic and geometric structures of hetero-interface, we summarized six basic characteristics of hetero-interface: electronic structure modulation, charge separation, electron transport, support effect, defect effect, and synergic effect (Figure 5). In terms of electronic structure modulation, different substances possess distinct electronic structures. When two or more substances interacts with each other, the band structure and Fermi level of them will be changed and a new electronic structure will be formed at the hetero-interface [33,46]. Similarly, the difference in Fermi energy between two materials results in a space-charge region near the interface, so that influence the charge transport and separation process at the interface [81,82]. Furthermore, the formed interface changes the way of electron transfer, the electrons migrate more easily across the interface [69]. In addition to these electron-related features, hetero-interfaces also have some geometrically related characteristics. Generally, a hetero-interface possess the ability of supporting and stabilizing the active catalyst, this is the support effect of hetero-interface [87–91]. For example, some low-dimensional (0D) catalysts are usually supported on higher-dimensional materials (1D, 2D and 3D), thereby achieving full utilization of active sites and strong mechanical stability. A mismatch between two different materials would generate abundant lattice defects and stress at the contacted interface [66]. Moreover, the vacancies and heteroatom adulteration will inevitably occur at the hetero-interfaces [84–88]. These lattice defects are defined as defect effect of hetero-interfaces. It has been demonstrated that defect effect could alter the electronic structure and spatial charge distribution, thus adjusting the activity of water splitting and providing abundant active sites for water splitting [92–95]. Apart from above mentions, the synergistic effect is another important feature of hetero-interfaces. Different materials have different effects on the water splitting process. The formation of a hetero-interface may cause synergistic effect due to the different facilitating effects of the two (or more) materials that make up the interface [29,57,96–100].

**Figure 6.**
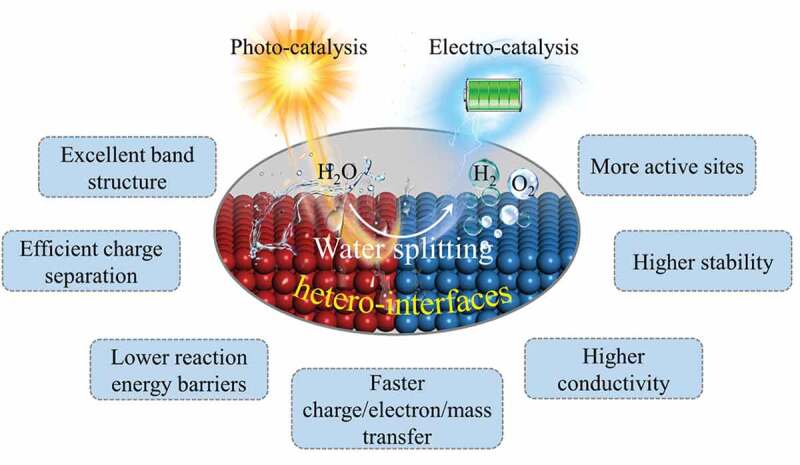
The roles of nanoscale hetero-interfaces in photo/electrocatalytic water splitting.

Nanoscale hetero-interface plays a crucial role in water splitting. In Figure 6, we summarized some significant functions of hetero-interface in water splitting. Through constructing the hetero-interfaces, the inherent catalytic properties of individual catalyst can be significantly changed, which endow the compound photo- and electro-catalysts with excellent water splitting property. At first, constructing the hetero-interfaces can modify the electronic structure of catalyst, such as *d* band center and the density of states (DOS) near the Fermi level, to optimize the adsorption and desorption of intermediates and reduce the reaction energy barriers [69,101–104]. Apart from turning the electronic structure to improve the intrinsic activity of catalysts, constructing the hetero-interfaces can also extend the catalytically active sites to create more reaction sites for water splitting [47,71,105]. Furthermore, hetero-interfaces have more channels at the interface for charge, electron and mass transfer, thereby improving the kinetics of the reaction [106–109]. The strong contact surfaces between the different components increase both the mechanical stability and the electrical conductivity of the system [110,111]. To sum up, benefitting from their unique characteristics, hetero-interfaces present great advantages in photo- and electrocatalytic water splitting, which can optimize the electronic structure, lower the reaction energy barriers, realize an efficient charge separation process and a fast electron/mass transfer process. Meanwhile, the formed hetero-interfaces enable the composite catalysts to possess more active sites, higher electron conductivity and mechanical stability.

## 3. The progress of hetero-interfaces for photo/electrocatalytic water splitting

At present, the construction of hetero-interfaces by integrating different catalysts has been extensively investigated to improve the efficiency of water splitting, such as metal-metal, metal-metallic compounds (e.g. oxide, hydroxide, phosphate, chalcogenide, nitride and carbide), and metal-nonmetal (e.g. graphene, black phosphorus, and polymers) hetero-interfaces [37,39,40,42,82]. These heterojunctions possess different characteristics that promote water splitting in different ways. We will systematically review the research progress of photocatalytic and electrocatalytic catalysts with hetero-interfaces for water splitting, according to the characteristics of them.

### 3.1 Electronic structure modulation

**Figure 7.**
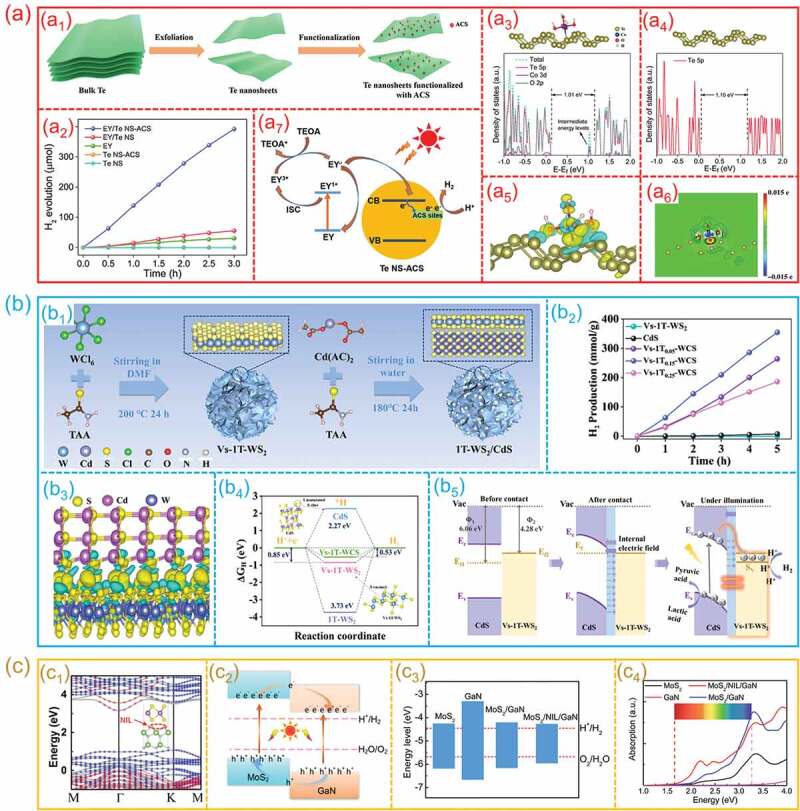
**The electronic structure modulation of nanoscale hetero-interfaces for photocatalytic water splitting. (a) ACS/Te NS hetero-interfaces**: (a1) the illustration of preparation process; (a2) Photocatalytic H_2_ generation performance; (a3, a4) the optimized atomic structure models, and the corresponding calculated DOS; (a5, a6) the charge distributions; (a7) Proposed mechanism of photocatalytic H_2_ evolution. (Reproduced with permission from [112], Copyright 2020, Wiley) **(b) CdS/WS**_2_ **hetero-interfaces**: (b1) the illustration of preparation process and corresponding atomic structure models; (b2) the performance of photocatalytic H_2_ evolution; (b3) the differential charge density; (b4) the calculated reaction energy barrier diagrams of H adsorption; (b5) the energy band structure of hetero-interfaces and the mechanism of photocatalytic hydrogen evolution on hetero-interfaces. (Reproduced with permission from [113], Copyright 2022, Elsevier) **(c) MoS**_2_**/GaN hetero-interfaces**: (c1) Energy band structures; (c2) Schematic of the charge-transfer; (c3) Band edge positions; (c4) Calculated absorption spectra. (Reproduced with permission from [104], Copyright 2018, American Chemical Society).

The electron redistribution and the difference of Fermi level on contacted interfaces lead to the changes of electronic structure at the hetero-interfaces. Different substances have different band structures and Fermi levels, when they connect with each other, a unique electronic structure will appear at the connected interface. The change of electronic structure affects the activity of catalysts. The performance of photo- and electro-catalytic water splitting can be enhanced through constructing the rational hetero-interfaces to optimize the electronic structure of composite catalysts. For instance, an atomic Co species anchored 2D Te nanosheets (ACS/Te NS) hetero-interface was fabricated by exfoliation and functionalized assembly (Figure 7a_1_) [112]. As presented in Figure 7a_2_, the ACS/Te NS hetero-interfaces exhibit a more stable and higher photocatalytic H_2_ production process than that of pristine Te NS. As displayed in Figures 7a_3_ and a_4_, compared to Te NS, the total density of states (DOS) of ACS/Te NS demonstrates an increasing shift of the VBM toward the Fermi level, which results in a shorter bandgap and easier excitation of charge carriers. From the distributions of charge density at the ACS/Te NS interface, it can be found that the ACS/Te NS interface has a higher charge density, indicating that ACS and Te NS have a strong mutual interaction, and the charge transfer from Te NS to ACS (Figures 7a_5_,a_6_). The enhanced performance of ACS/Te NS hetero-interfaces can be attributed to the optimization of electronic structure (Figure 7a_7_). Moreover, a 1T-WS_2_/CdS hetero-interface with S vacancies was designed for high-efficiency photocatalytic HER [113]. Figure 7b_1_ shows the fabrication process. The as-prepared 1T-WS_2_/CdS hetero-interface achieved 70.9 mmol/g/h hydrogen evolution rate, as the Figure 7b_2_ exhibited. Compared with individual S vacancies 1T-WS_2_ and CdS, S vacancies 1T-WS_2_/CdS hetero-interface presents an obviously enhanced photocatalytic performance. S vacancies in 1T-WS_2_ induce the electrons migration from CdS to S vacancies to produce an enhancement of field strength at hetero-interface to boosting photocatalytic hydrogen evolution. The enhanced photocatalytic performance can be attributed the modulation of electronic structure and facilitation of *H adsorption on 1T-WS_2_/CdS hetero-interface. Obviously, constructing the hetero-interfaces can optimize the electronic structure of catalysts, thus can perfect the adsorption of reaction intermediates and promote charge separation and transfer, finally realizing an efficient photocatalytic process (Figure 7b_3_-b_5_). In addition, the improvement of electronic structure, such as band structure adjustment, an enhance the absorption of light, thereby improving the photocatalytic efficiency. For instance, Zhang et al. investigated the structural, electronic, and optical properties of MoS_2_/GaN hetero-surface by theoretical calculations and experimental analysis [104]. As shown in Figure 7c_1_, the MoS_2_/GaN hetero-interfaces have an indirect band gap, which benefits the photoexcited carriers’ lifetime. Meanwhile, after nitridation treatment, the conduction band edge and valence band edge of the MoS_2_ side increase. The enhanced CBM may lead to greater electron accumulating capabilities on the GaN side (Figure 7c_2_). The MoS_2_/GaN heterostructure with a special interface exhibits a strong optical absorption ability and excellent band structure, suggesting that MoS_2_/GaN heterostructure can be employed as a feasible photocatalyst for the generation of hydrogen (Figures 7c_3_ and c_4_).

**Figure 8.**
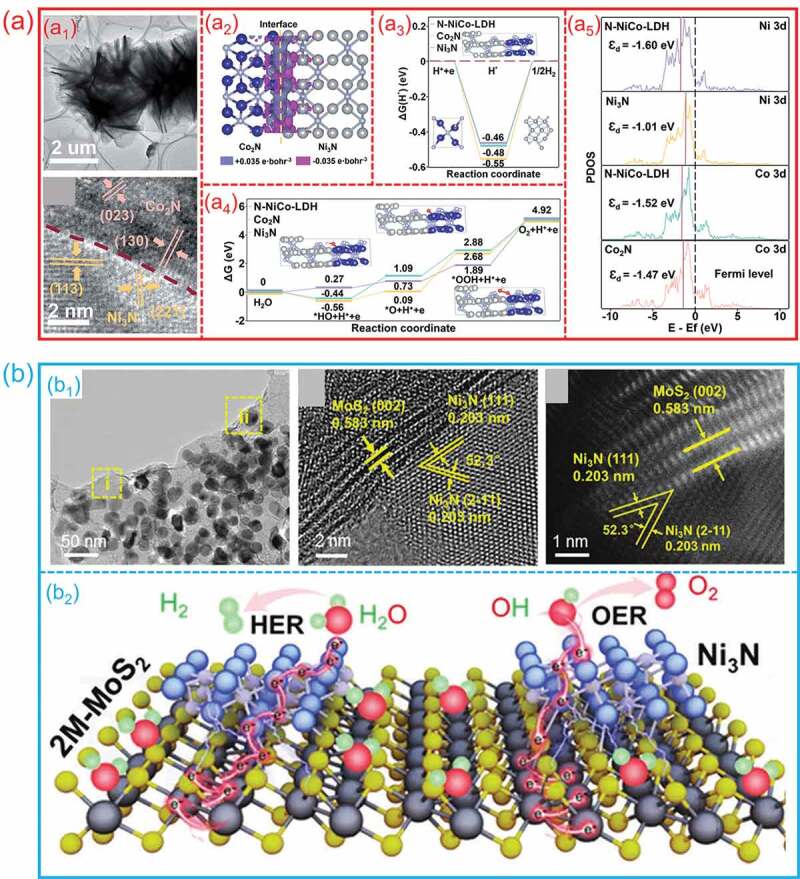
**The electronic structure modulation of nanoscale hetero-interfaces for electrocatalytic water splitting. (a) Ni**_3_**N/Co**_2_**N hetero-interfaces**: (a1) TEM and HRTEM images; (a2) the charge density distribution on hetero-interfaces. (a3) the calculated free energy changes for the HER process on the surface of Ni_3_N/Co_2_N, Ni_3_N, and Co_2_N. (a4) the calculated free energy changes for the OER process on the surface of Ni_3_N/Co_2_N, Ni_3_N, and Co_2_N. (a5) the density of states of Ni and Co 3d orbitals for Ni_3_N/Co_2_N, Ni_3_N, and Co_2_N. (Reproduced with permission from [28], Copyright 2021, Royal Society of Chemistry) **(b) Ni**_3_**N/MoS**_2_ **hetero-interfaces**: (b1) TEM and HRTEM images; (b2) the possible mechanism of water splitting process on the Ni_3_N@2 M-MoS_2_ hetero-interfaces. (Reproduced with permission from [68], Copyright 2022, Wiley).

For design of electrocatalysts, the favorable electronic structure can enhance the reaction kinetics and optimize the catalytic reaction pathways to improve the activity. Constructing hetero-interfaces between different catalysts can efficiently regulate the electronic structure of electrocatalysts, which lowering the reaction energy barriers and improve the intrinsic activity. For example, Wen et al. synthesized a bifunctional hetero-interface between Ni_3_N and Co_2_N, which shows an optimized interfacial electronic structure and reduced the energy barrier for HER/OER, so that to improve the activity [28]. As shown in Figure 8a_1_, the transmission electron microscopy (TEM) and high-resolution transmission electron microscopy (HRTEM) images reveal that the unique flower-like hierarchical architecture of the Ni_3_N/Co_2_N hetero-interface is beneficial for exposing the active site. Because of the redistribution of electrons at the Ni_3_N/Co_2_N hetero-interface, the energy barrier for both HER and OER can be lowered. (Figures 8a_2_-a_4_). The calculated electronic structures demonstrate that the formed hetero-interface changes the d-band center of the metal atoms near the interface with a slight downshifting, reducing the occupation of the anti-bonding states for the metal-intermediates and thus improving their catalytic properties (Figures 8a_5_). This work demonstrates the importance of the hetero-interface in the aggregation of interface electrons, modulation of electronic structure, and enhancement of catalytic activity. Wu et al. design a bifunctional electrocatalyst of Ni_3_N/MoS_2_ hetero-interface to realize a highly efficient water electrolysis process at a high current density [68]. Figure 8b_1_ shows the TEM and HRTEM images of Ni_3_N/MoS_2_ hetero-interfaces, Ni_3_N was uniformly inlaid in MoS_2_ nanosheets and formed a clear interface. The desired conductivity of Ni_3_N/MoS_2_ heterointerface provide a high-speed channel for electron and mass transfer (Figure 8b_2_). Moreover, the electronic states of Ni and N atoms around the Fermi-level was regulated through forming Ni_3_N/MoS_2_ heterointerface, which can optimize the free energies of reaction intermediates. The outstanding electrocatalytic performance and excellent durability of the designed Ni_3_N/MoS_2_ hetero-interface suggest that it is a promising electrocatalyst for large-current alkaline water electrolysis.

### 3.2 Charge separation

**Figure 9.**
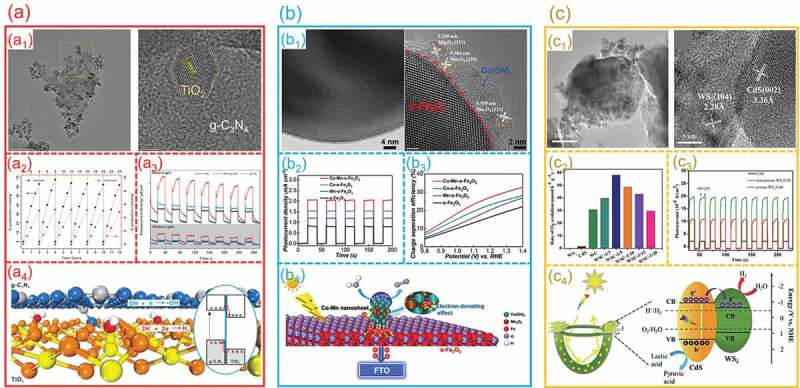
**The charge separation on hetero-interfaces for photocatalytic water splitting. (a) TiO**_2_**/g-C**_3_**N**_4_ **hetero-interfaces**: (a1) TEM and HRTEM images; (a2) Photocatalytic H_2_ generation performance; (a3) Transient photocurrent curves; (a4) Mechanism of Photocatalytic H_2_ Production. (Reproduced with permission from [114], Copyright 2021, American Chemical Society) **(b) Co-Mn/Fe**_2_**O**_3_ **hetero-interfaces**: (b1) TEM and HRTEM images; (b2) Transient photocurrent curves; (b3) charge separation efficiencies; (b4) Schematic electron transfer paths. (Reproduced with permission from [115], Copyright 2019, Wiley) **(c) WS**_2_**/CdS hetero-interfaces**: (c1) TEM and HRTEM images; (c2) Photocatalytic H_2_ evolution performance; (c3) Transient photocurrent curves; (c4) Mechanism schematics for photocatalytic H_2_ evolution. (Reproduced with permission from [116], Copyright 2020, Elsevier).

High efficiency of photoinduced charge-carrier separation is the key factor to determine the photocatalytic activity of photocatalysts. The photoinduced charge-carrier-separation efficiency can be enhanced by constructing a hetero-interface between photocatalysts. To achieve effective photocatalytic activity on a hybrid catalyst, two factors are important: easy transfer of photogenerated carriers at the hetero-interface and their quick reactivity with redox species on the surface of photocatalyst. Using a simple hydrothermal approach, Feng et al. from our group synthesized a TiO_2_/g-C_3_N_4_ hetero-interface with a tightly coupled Ti−N−C bond at the interface for photocatalytic H_2_ evolution [114]. As presented in Figure 9a_1_, the TEM and HRTEM images of TiO_2_/g-C_3_N_4_ composite show that TiO_2_ nanoparticle is combined with g-C_3_N_4_ nanosheet and formed a clear hetero-interface. This hetero-interface exhibits a highly efficient activity for H_2_ generation, which is much higher than that of pure g-C_3_N_4_ (Figure 9a_2_). The transient photocurrent curves manifest that the TiO_2_/g-C_3_N_4_ hetero-interface are favorable for charge separation and transfer (Figure 9a_3_). According to these experimental results, the formed TiO_2_/g-C_3_N_4_ hetero-interface can effectively enhance the separation and transfer of the photogenerated carriers to accelerate the hydrogen evolution (Figure 9a_4_). Moreover, Yang et al. constructed a Co-Mn nanosheets/Fe_2_O_3_ hetero-interface [115], as shown in Figures 9b_1_ of HRTEM images. The optimized Co-Mn/Fe_2_O_3_ hetero-interface exhibited an excellent photocatalytic activity, as well as a remarkable stability. As shown in Figures 9b_2_ and **b**_3_, Co-Mn/Fe_2_O_3_ hetero-interface can significantly enhance the ability of charge transfer and separation. They observed that introducing the Mn site onto the nanosheets may induce electron donation to the Co site and assist the activation of the OH group, significantly increasing the inherent photocatalytic activity (Figure 9b_4_). Furthermore, this deposition approach might be used in composites with other metals, making it a flexible and promising strategy for decorating photocatalysts with other metallic materials. Similarly, Su et al. in our group have been successfully fabricated a porous WS_2_/CdS hetero-interface for photocatalytic HER [116]. From the TEM and HRTEM in Figure 9c_1_, a well-defined hetero-interface can be found between WS_2_ and CdS. As shown in Figure 9c_2_, the formed WS_2_/CdS hetero-interface exhibits a better photocatalytic activity than that of pure CdS. The transient photocurrent curves in Figure 9c_3_ reveals that the hetero-interface arrangement facilitated the transport of photo-excited electrons while slowing electron and hole recombination, which leading to an intensive light absorption. This work demonstrated that the well-defined hetero-interface and thin lamellar structure functioned synergistically to facilitate the transport of photo-excited electrons and slow electron and hole recombination can achieve a high-efficiency H_2_ evolution activity (Figure 9c_4_).

**Figure 10.**
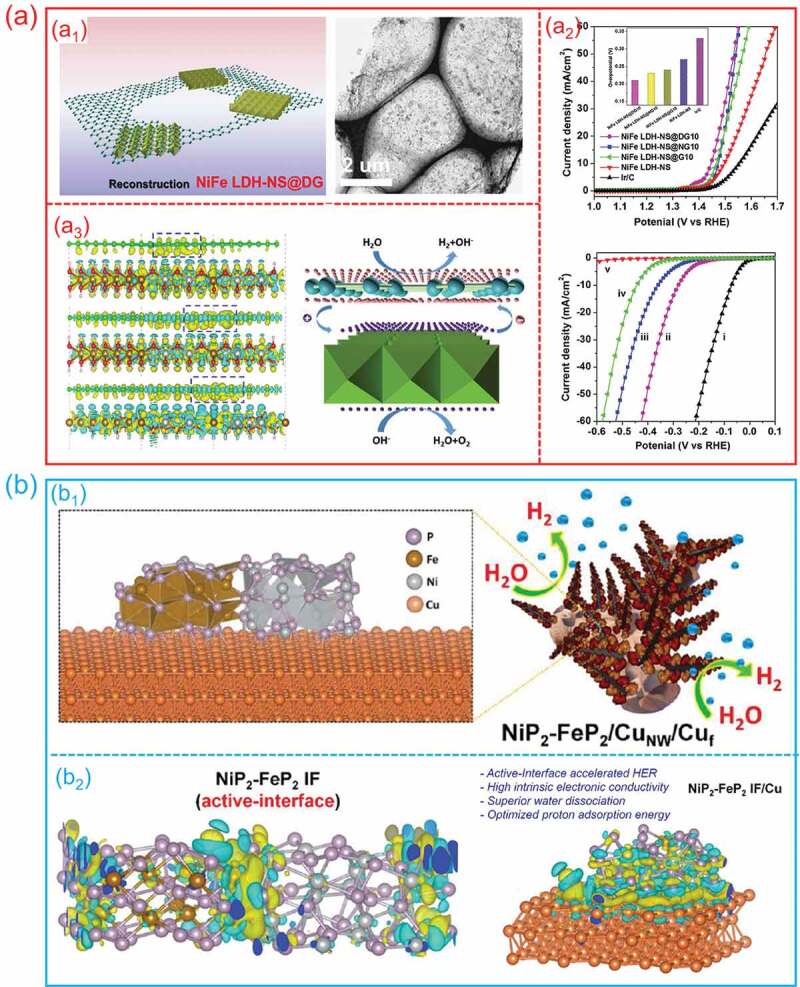
**The charge separation on hetero-interfaces for electrocatalytic water splitting. (a) NiFe-LDH/DG hetero-interfaces**: (a1) structure model and TEM image; (a2) OER and HER performance; (a3) the charge density distribution and the probable electrocatalytic water splitting process. (Reproduced with permission from [33], Copyright 2017, Wiley) **(b) NiP**_2_**/FeP**_2_**/Cu hetero-interfaces**: (b1) the structure model; (b2) the charge density difference plot. (Reproduced with permission from [117], Copyright 2021, American Chemical Society).

For electrocatalysis, charge separation mainly refers to the charge density difference at the hetero-interfaces, which can influence the adsorption of intermediates and accelerate the transportation of electrons at the interface, thus realizing an enhanced electrocatalytic activity. For example, Jia et al. assembled a bifunctional NiFe-layered double hydroxide and defective graphene (NiFe-LDH/DG) hetero-interface for water splitting by electrostatic self-assembly method. The formed NiFe-LDH/DG hetero-interface not only highlights the high OER activity of FeNi-LDH, but also presents the remarkable HER enhancement due to the charge aggregation around the hetero-interface [33]. Figure 10a_1_ shows the structure model and TEM image of NiFe-LDH/DG hetero-interfaces. Figure 10a_2_ shows the OER and HER properties of as-prepared samples. Obviously, the NiFe-LDH/DG hetero-interface shows a more remarkable activity and stability. Because of the charge separation between NiFe LDH and DG (Figure 10a_3_), leading to significantly enhanced density of electron transfer at hetero-interfaces, NiFe LDH/DG hetero-interface shows an outstanding OER and HER performance.

Motivated by theoretical predictions, Kumar et al. employed a facile strategy to prepare an interface-rich NiP_2_/FeP_2_/Cu hybrid catalyst [117]. Figure 10b_1_ shows the structure model of NiP_2_/FeP_2_/Cu hetero-interfaces. They discovered that when NiP_2_/FeP_2_ is coupled with metallic Cu, the electronic structure and localized charge density at the heterointerface can be greatly modified, leading in optimal proton adsorption energy and a lower barrier for water dissociation, hence improving alkaline HER (Figure 10b_2_). The developed catalyst displayed remarkable alkaline HER activity requiring a low overpotential of 23.6 mV at −10 mA/cm^2^, outperforming the state-of-the-art Pt due to greater intrinsic activity, conductivity, and a large number of active sites.

### 3.3 Electron/charge/mass transfer

**Figure 11.**
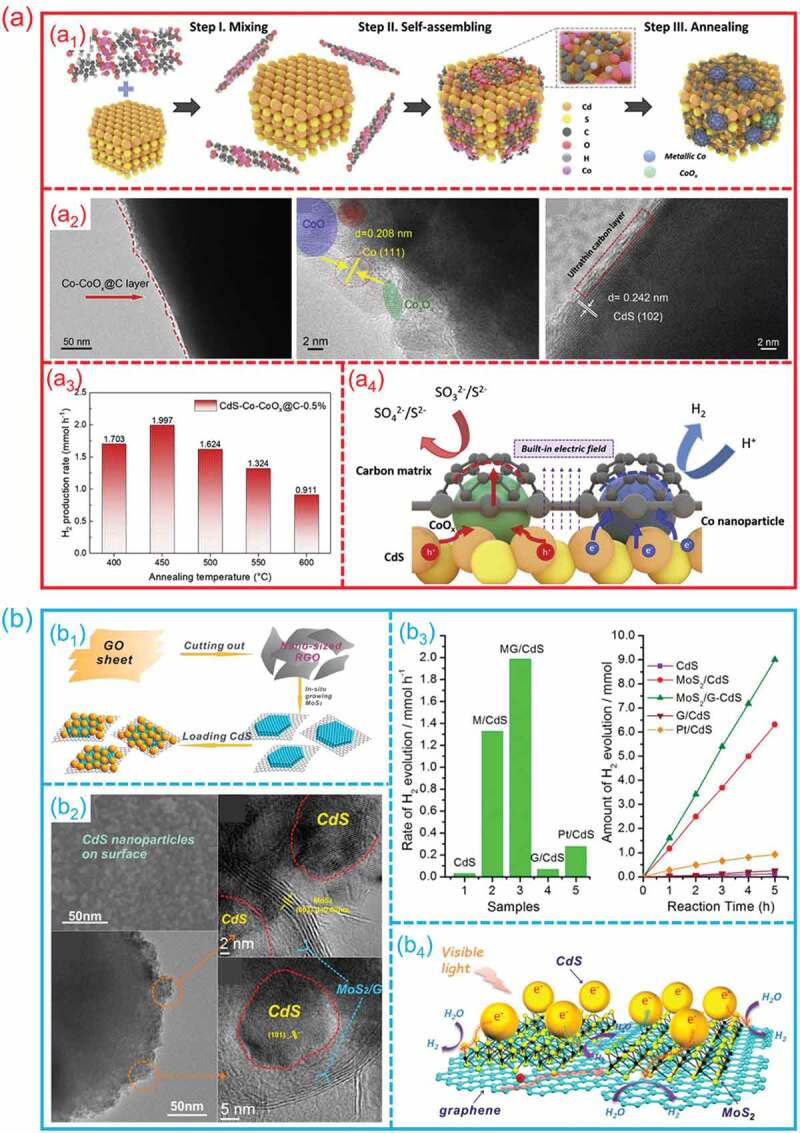
**The electron/charge/mass transfer on hetero-interfaces during photocatalytic water splitting. (a) Co-CoO**_x_**/C/CdS hetero-interfaces**: (a1) Schematically illustration of preparation; (a2) TEM and HRTEM images; (a3) Photocatalytic hydrogen evolution performance; (a4) the charge transfer process. (Reproduced with permission from [118], Copyright 2021, Elsevier) **(b) MoS**_2_**/Graphene/CdS hetero-interfaces**: (b1) Schematically illustration of fabrication; (b2) TEM and HRTEM images; (b3) Photocatalytic hydrogen evolution performance; (a4) the charge carriers transfer and separation on hetero-interface. (Reproduced with permission from [119], Copyright 2014, American Chemical Society).

The hetero-interfaces provide a fast channel for electron/charge/mass transport due to the abundant lattice defects and the difference in charge density at the interface, which can accelerate the kinetic process of catalytic reaction. For example, as shown in Figure 11a, Ren et al. induced the dual cobalt active species (Co and CoOx) on the surface of CdS to produce a hetero-interface for high-efficiency photocatalytic H_2_ generation [118]. Figure 11a_1_ shows the fabrication process of Co-CoO_x_/C/CdS hetero-interfaces, TEM and HRTEM images show that the Co-CoO_x_ species were formed on the surface of CdS (Figure 11a_2_). On the Co-CoO_x_/C/CdS hetero-interfaces, photocatalytic H_2_ production performance was improved about 12.5-fold (Figure 11a_3_). The carbon matrix and co-existing dual cobalt active species significantly increase charge carrier movement and photogenerated electron-hole separation for improved photocatalytic activity (Figure 11a_4_). Chang et al. fabricated a MoS_2_/G-CdS heterostructure for photocatalytic H_2_ production (Figure 11b_1_) [119]. Figure 11b_2_ show the scanning electron microscopy (SEM), TEM and HRTEM images of MoS_2_/G-CdS composite, it can be seen that a will defined hetero-interfaces was formed between MoS_2_ and G-CdS. The MoS_2_/G-CdS heterostructure presents the best photocatalytic H_2_ generation activity (Figure 11b_3_). It is proved that, owing to the specific connected interface, the photo-induced electrons can easily transfer graphene substrate to participate in reactions. The addition of graphene is thought to improve charge transfer abilities and slow the recombination of electron-hole pairs, therefore increasing photocatalytic H_2_ evolution activities (Figure 11b_4_).

**Figure 12.**
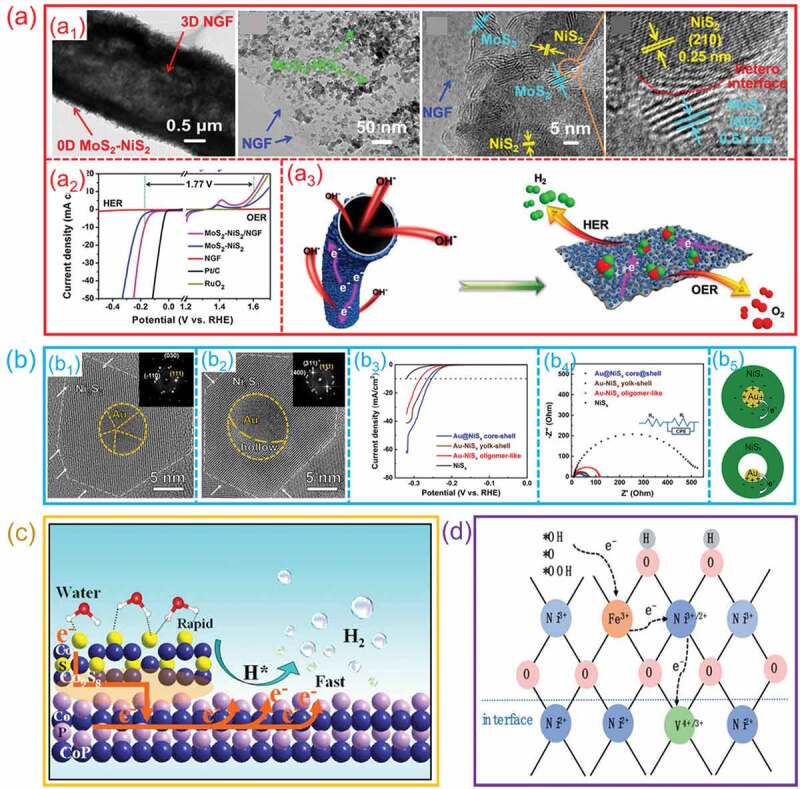
**The electron/charge/mass transfer on hetero-interfaces during electrocatalytic water splitting. (a) MoS**_2_**/NiS**_2_ **hetero-interfaces**: (a1) TEM and HRTEM images; (a2) the activity of overall water splitting; (a3) Schematic diagram of electron/mass transport process and the mechanism of water splitting. (Reproduced with permission from [73], Copyright 2019, Elsevier) **(b) Au/NiS**_x_ **hetero-interfaces**: (b1, b2) HRTEM images of core-shell and yolk-shell structures; (b3) HER performance; (b4) Nyquist plots; (b5) the electron transfer process on core-shell and yolk-shell hetero-interfaces. (Reproduced with permission from [120], Copyright 2020, Elsevier) **(c)** the catalytic mechanism of CoP/Co_9_S_8_ hetero-interfaces. (Reproduced with permission from [121], Copyright 2019, American Chemical Society) **(d)** Proposed mechanism for electron transfer on NiFev/nifevn hetero-interfaces. (Reproduced with permission from [72], Copyright 2022, Royal Society of Chemistry).

Electrochemical water splitting is heavily reliant on electron/charge/mass movement and active sites; nevertheless, the difficulties in enabling electron/charge/mass transport and exposing enough active sites is a key barrier for both halves of the entire water splitting. To address these two issues, Kuang et al. developed a simple and cost-effective method to create a bimetallic sulfide-anchored MoS_2_-NiS_2_/NGF hetero-interface for electrocatalytic overall water splitting [73]. As shown in Figure 12a_1_, the SEM, TEM and HRTEM images of as-prepared sample show that 0D MoS_2_ and NiS_2_ were grown on nitrogen-doped graphene foam (NGF), and hetero-interfaces were formed between them. The MoS_2_-NiS_2_/NGF hetero-interface has a much lower overpotential and charge transfer resistance than other samples (Figure 12a_2_). The MoS_2_-NiS_2_/NGF hetero-interface provides abundant active sites and diverse pathways for electron transfer. As a consequence of the strong contacts between MoS_2_/NiS_2_ nanoparticles and NGF with a high-speed channel for mass and electron transfer, which improved the performance towards HER and OER (Figure 12a_3_). Lv et al. designed and synthesized an Au-NiS_x_ hetero-interfaces with core@shell and yolk-shell structures [120]. Figures 12b_1_ and 12b_2_ show the HRTEM images of Au/NiS_x_ core@shell and Au-NiS_x_ yolk-shell hetero-interfaces, respectively. Catalytic performance tests show that Au/NiS_x_ core@shell hetero-interfaces outperform yolk-shell hetero-interfaces and “pure” NiS_x_ NPs in terms of HER property (Figure 12b_2_). The electrochemical impedance measurements show that the Au/NiS_x_ core@shell hetero-interfaces possess smaller resistance for electron transfer (Figure 12b_3_). Benefitting from the unique hetero-interface structure, the electron transfer between Au and NiS_x_ was fast on the Au/NiS_x_ core@shell hetero-interfaces, so that inducing an enhanced HER performance (Figure 12b_4_). In addition, Meng et al. prepared a CoP/Co_9_S_8_ hetero-interface by performing an in-situ transformation on Co_9_S_8_, resulting in the production of an atomic hetero-interface (Figure 12(c)). Experiments and theoretical studies show that the as-prepared atomic hetero-interface may produce a local charge distribution on interface, which not only speeds up the charge transfer but also optimizes the adsorption energy of intermediates in favor of the quick H_2_ evolution [121]. At the same time, the CoP/Co_9_S_8_ atomic hetero-interface can also enhance the capability of water adsorption. Taking use of these exceptional benefits, an alkaline electrolyzer with CoP/Co_9_S_8_ atomic hetero-interface as both electrodes achieves a remarkable overall water splitting property. Lu et al. built an elaborate NiFeV/NiFeVN hetero-interface to show how the nanoalloy/nitride heterointerfaces might improve the OER performance (Figure 12(d)). The variable-valence V^3+/4+^ electron acceptor at the core@shell interface and the heterojunction effect in the NiFeV/NiFeVN core successfully accelerated electron transport from the OER intermediates through the amorphous NiFeOOH shell [72]. As a consequence, both the OER and HER process can be improved due to the fast electron transport on the NiFeV/NiFeVN hetero-interface.

### 3.4 Support effect

**Figure 13.**
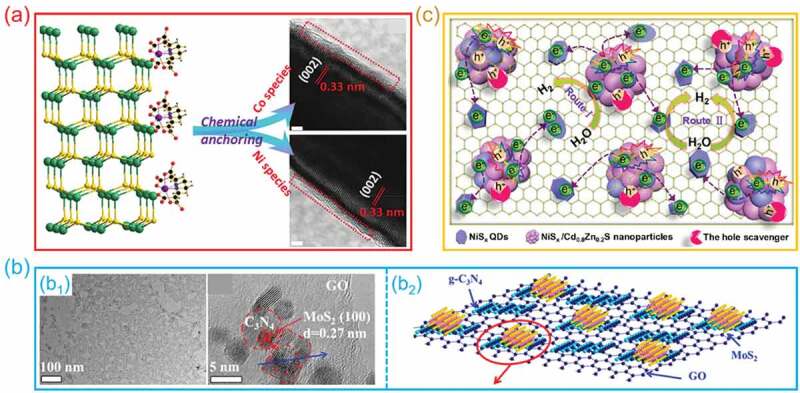
**The support effect of hetero-interfaces for photocatalytic water splitting. (a)** the structure diagram and HRTEM images of Co-Ni Species/CdS hetero-interfaces. (Reproduced with permission from [122], Copyright 2017, Wiley) **(b) MoS**_2_**/g-C**_3_**N**_4_**/GO hetero-interfaces**: (b1) TEM and HRTEM images; (b2) the structure diagram. (Reproduced with permission from [123], Copyright 2017, American Chemical Society) **(c)** the possible charge transfer process and the catalytic reaction mechanism on NiS/Cd_0.8_Zn_0.2_S/rGO hetero-interfaces. (Reproduced with permission from [110], Copyright 2018, American Chemical Society).

Constructing a hetero-interface can not only modulate the electronic structure, improving the intrinsic activity of the catalysts, but also can expose more active sites of the catalysts through dispersion, and lead to a higher conductivity. Moreover, the strong bond between different catalysts can prevent the active species from agglomerating, debarking, corroding, and structural destroy during the reaction process, which can effectively improve the stability of catalyst. All these advantages can be attributed to the support effect of the hetero-interfaces. For instance, Zhao et al. proposed an innovative and straightforward method to anchor well-dispersed metal (Co and Ni) species on the surface of CdS to form a Co-Ni Species/CdS hetero-interface [122], As shown in Figure 13(a). The Co, Ni species/CdS hetero-interfaces presented an enhanced photocatalytic H_2_ evolution activity with a H_2_ generation rate of 4.3 mmol h^−1^, which is even exceeding that of Pt-loaded CdS. Co-Ni Species/CdS hetero-interface also presents an outstanding stability, the performance is rather stable without apparent decay during the six cycling tests, which is much more stable than that of CdS loaded with Pt. Further investigations suggested that, as active species, the Co/Ni dispersed on CdS surface not only enhance the utilization of active sites, but also improve the stability of them, so that achieving an outstanding performance for photocatalytic H_2_ generation. This work demonstrates that constructing the hetero-interfaces can effectively expose the active sites and enhance the speed of charge transport. Moreover, An effective photocatalyst for hydrogen evolution was developed by Wang and colleagues by the construction of a MoS_2_/g-C_3_N_4_/graphene oxide (GO) tripartite hetero-interfaces [123]. As shown in Figure 13b_1_, TEM and HRTEM images show that the g-C_3_N_4_ and MoS_2_ quantum dots (QDs) were uniformly mounted on GO and formed a MoS_2_/g-C_3_N_4_/GO hetero-interface. Compared with MoS_2_/g-C_3_N_4_ and MoS_2_ QDs, the photocatalytic H_2_ evolution property of MoS_2_/g-C_3_N_4_/GO hetero-interface is greatly increased. The H_2_ production rate for MoS_2_/g-C_3_N_4_ sis1.06 mmol h^−1^ g^−1^, which is about 4.3 times higher than that of MoS_2_ QDs (0.20 mmol h −1 g − 1). When combining with GO, the H_2_ production rate for tripartite MoS_2_/g-C_3_N_4_/GO hetero-interface is about 1.5 times higher than that of MoS_2_/g-C_3_N_4_. Obviously, GO further enhanced the photocatalytic activity of HER. In this tripartite hetero-interface, the formed MoS_2_/g-C_3_N_4_ interface facilitates the migration of photogenerated electron−hole pairs. GO mainly acts as the hole transport layer, achieving the efficient transfer of collected carriers in g-C_3_N_4_ (Figure 13b_2_). Similarly, Xue et al. fabricated a NiS/Cd_0.8_Zn_0.2_S/reductive graphene oxide (rGO) hetero-interface to enhance the electron transfer (Figure 13(c)). They concluded that introducing the high density of NiSx QDs onto rGO nanosheet surface can improve the conductivity of interface and changed the electron distributions on the interface, consequently inducing an enhanced photocatalytic performance. The above research results show that the support effect of hetero-interfaces plays a significant role in photocatalytic water splitting, which can supply more active sites to adsorb reactants and enhance the transportation and separation of photogenerated charge-carriers.

**Figure 14.**
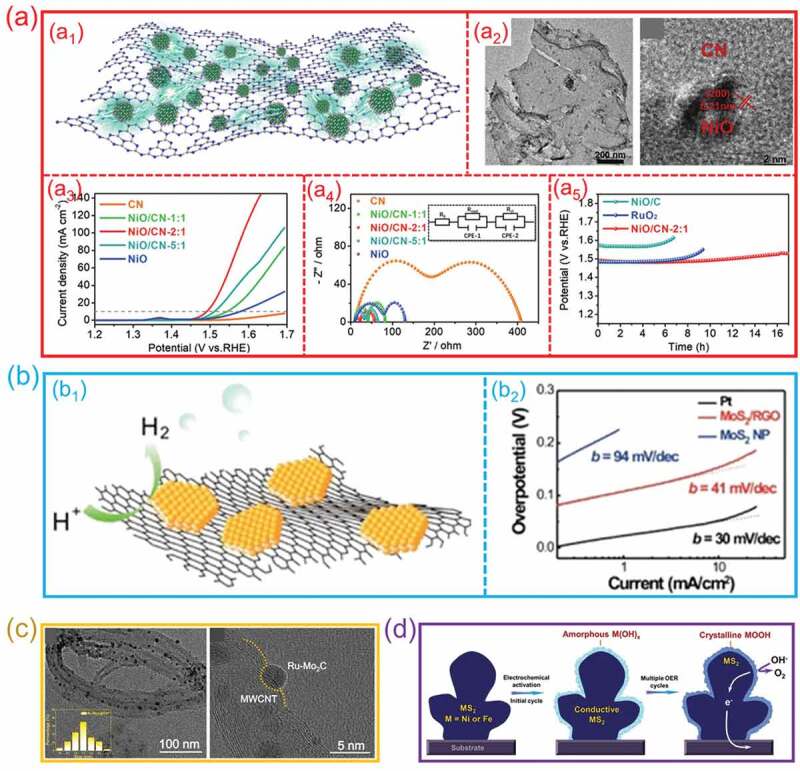
**The support effect of hetero-interfaces for electrocatalytic water splitting. (a) NiO/g-C**_3_**N**_4_ **hetero-interfaces**: (a1) the s structure diagram; (a2) TEM and HRTEM images; (a3) OER performance; (a4) Nyquist plots; (a5) the stability of NiO/g-C_3_N_4_ hetero-interfaces in 1 M KOH. (Reproduced with permission from [124], Copyright 2019, Wiley) **(b) MoS**_2_**/rGO hetero-interfaces**: (b1) the structure diagram; (b2) Tafel slopes. (Reproduced with permission from [125], Copyright 2011, American Chemical Society) **(c) Ru-Mo**_2_**C/CNT hetero-interfaces**: TEM and HRTEM images. (Reproduced with permission from [71], Copyright 2021, Nature) **(d) Ni(Fe)OOH/Ni(Fe)S**_2_ **hetero-interfaces**: the formation process of hetero-interface and the mechanisms of enhanced OER. (Reproduced with permission from [126], Copyright 2017, Royal Society of Chemistry).

During electrocatalytic water splitting, the support effect of hetero-interfaces not only expose abundant active sites for reactant adsorption, but also enhance the conductivity of catalysts for electron transport. More importantly, it can significantly improve the stability of electrocatalysts. In our previous studies, we constructed a NiO/polymer carbon nitride (NiO/CN) hetero-interface with high conductivity for efficient electrocatalytic OER (Figure 14a_1_) [124]. As the TEM and HRTEM images shown in Figure 14a_2_, the NiO nanocrystals was uniformly grown on g-C_3_N_4_ nanosheets. The NiO/g-C_3_N_4_ hetero-interface exhibits much-enhanced OER activity than that of pristine NiO and g-C_3_N_4_ (Figure 14a_3_). The electrochemical impedance analysis (Figure 14a_4_) shows that the NiO/g-C_3_N_4_ hetero-interface has a high conductivity due to the formation of metallic Ni – N bond. The theoretical calculations suggest that NiO/g-C_3_N_4_ hetero-interface with Ni – N bond exhibits a reduced reaction energy barriers for the adsorption of intermediates. This highly conductive hetero-interface not only increases the speed of charge transport but also promotes the mass transfer in OER. Moreover, NiO/g-C_3_N_4_ presents more perdurable stability than that of NiO (Figure 14a_5_), which can be attributed to the strongly mechanical stability of g-C_3_N_4_ during electrolysis. Because of the highly conductive and stable metal-nitrogen interface, this compound possesses an efficient OER. Moreover, Li et al. used rGO as a substrate to composite with MoS_2_ to obtain a MoS_2_/rGO hetero-interface (Figure 14b_1_) [125]. The MoS_2_/rGO hetero-interface presents a superior electrocatalytic HER activity compared with MoS_2_. A very low Tafel slope of ∼41 mV/decade for HER was obtained on MoS_2_/rGO hetero-interface (Figure 14b_2_), this considerably outperforms prior MoS_2_ catalysts. Because of the abundance of active sites on hetero-interface as well as the superior electrical connection to the graphene, MoS_2_/rGO hetero-interface shows an obvious enhancement in HER activity. Carbon nanotubes are also used as conductive substrates for compounding with catalysts [30,71,111,127]. Wu et al. used a facile microwave pyrolysis method to fabricate a Ru-Mo_2_C/carbon nanotube (CNT) hetero-interface for electrocatalytic HER [71]. TEM and HRTEM images (Figure 14(c)) show that a clear hetero-interface has been formed between Ru-Mo_2_C and CNT. Benefitting from the high conductivity of CNT, the Ru-Mo_2_C/CNT hetero-interface has a very low overpotential of 15 mV, and a huge turnover frequency value of up to 21.9 s^−1^ in 1.0 M KOH electrolyte. More importantly, the activity of Ru-Mo_2_C/CNT hetero-interface can be maintained for more than 1000 h without decay due to the stable chemical feature of CNT, which is much higher than that of Ru-Mo_2_C. Otherwise, MXene and MoS_2_ are usually used as substrates to combine with the active catalysts to enhance the property of electrocatalytic water splitting [54,124]. For instance, Xie et al. fabricated a FeS_2_/MXene hetero-interface to achieve an efficient water splitting process by exposing the active sites and improving the conductivity [128]. The FeS_2_/MXene hetero-interface also exhibits an excellent stability for overall water splitting with a negligible decay after 30 h, which probably benefits from the protective effect of the MXene substrate. Wei et al. reported a Ir/MoS_2_ hetero-interface, which also exhibits a high catalytic activity for overall water splitting in alkaline media due to the more Ir sites are exposed [50]. In addition, Zhou et al. found that a specific hetero-interface between oxyhydroxide and nickel – iron disulfide, which is formed through an in-situ electrochemical reaction (Figures 14(d)). Such a hetero-interface takes use of extremely catalytically active oxyhydroxide surfaces as well as the strong conductivity of the inner disulfide phase, resulting in a very low overpotential for OER [126]. The crystalline oxyhydroxide layer has the ability to effectively prevent the disulfide core from experiencing any further oxidation. Additionally, this layer is responsible for maintaining the core – shell structure of the catalyst, which is widely regarded as being essential for stable and effective OER performances. Obviously, the substrates with high conductivity and large surface area can availably accelerate the electron transfer and expose more active sites to promote the catalytic activity, the substrates with strong chemical stability can maintain the structure of active phase during reaction to enhance the catalytic stability. Therefore, combining catalysts with suitable substrates to produce a hetero-interface can effectively improve the catalytic performance through the support effect of interface.

### 3.5 Defect effect

**Figure 15.**
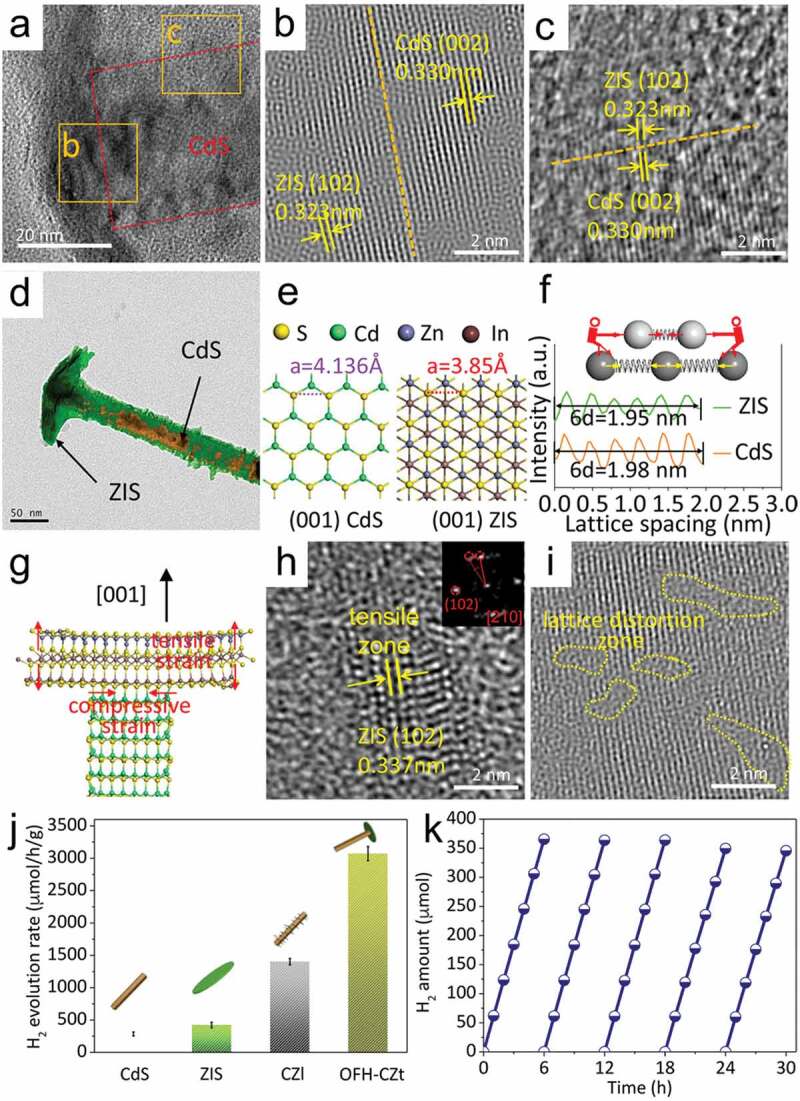
**The defect effect on ZnIn**_2_**S**_4_**/CdS hetero-interface for photocatalytic H**_2_ **evolution**. (a) TEM image; (b, c) HRTEM images; (d) the simulation image of CdS/ZnIn_2_S_4_ compounds; (e) the atomic models of CdS and ZnIn_2_S_4_; (f) the mechanism of intrinsic strain formation in hetero-interfaces; (g) Crystal boundary model of hetero-interface; (h) the lattice spacing measurement for ZnIn_2_S_4_; (i) the deformation of the lattice in the vicinity of the interface; (j, k) the performance of photocatalytic H_2_ evolution. Reproduced with permission from [129], Copyright 2020, Elsevier.

Due to the lattice mismatch and the different atomic species at the interface, there are often a large number of defects at the hetero-interfaces. These defects may effectively alter the electronic structure, facilitate electron transport, and offer an abundance of active sites, resulting in catalytic performance improvement. For instance, Zhu et al. synthesized a heterojunction photocatalyst with lattice strain defects (Figure 15) [129]. They obtained a ZnIn_2_S_4_/CdS hetero-interface by growing ZnIn_2_S_4_ nanosheets onto CdS nanowires. TEM and HRTEM images of ZnIn_2_S_4_/CdS composite (Figures 15(a)-(d)) shows the crystalline nature of ZnIn_2_S_4_/CdS hetero-interface and the lattice fringes of the ZnIn_2_S_4_/CdS hetero-interface near the grain boundary. It can be seen that a clear hetero-interface has been formed between ZnIn_2_S_4_ and CdS. The crystal models in Figures 15(e)-(g) show that the atom spacing of (001) plane in CdS is larger than that of ZnIn_2_S_4_. The production of Cd-S-In bonds results from a difference in the lattice spacing of CdS and ZnIn_2_S_4_ at the grain boundary. The lattice distortion at the interface has a significant impact on the structure of the ZnIn_2_S_4_/CdS hetero-interface, finally leading to edge tensile strain. The representative HRTEM images (Figures 15(h) and 15(i)) show that there have many tensile and lattice distortion zones at the hetero-interfaces. Benefiting from these interfacial defects, ZnIn_2_S_4_/CdS exhibits an enhanced photocatalytic H_2_ evolution activity (Figures 15(j) and 15(k)). The solid ZnIn_2_S_4_/CdS hetero-interfaces exhibited the highest H_2_ production rate, and presented a remarkable stability. The strong interaction between ZnIn_2_S_4_ and CdS and much interfacial defects accelerate the charge transfer on the hetero-interface, so that the electrons and holes easily transfer to CdS surface and ZnIn_2_S_4_ surface respectively to participate in redox reactions. A unique technique for guiding the design of the photocatalyst structure is given, which is advantageous for spatial charge transfer and separation. Ma et al. investigated the effect of interfacial strain engineering on the photocatalytic water splitting performance of C_2_N/In_2_SSe heterostructures through theoretical calculations [96]. They found the interfacial strain engineering can turning the electronic structure and band alignment, thereby optimizing the light absorption efficiency of the interface. Moreover, Zhang et al. found that the interfacial oxygen vacancies on Ti/Fe_2_O_3_ hetero-interfaces can markedly improve the charge separation and collection efficiencies, thus inducing a highly efficient photoelectrochemical water splitting process [93]. Kim et al. investigated the effect of oxygen vacancies at the Au/SrTiO_3_ interface on the photocatalytic water splitting performance [130]. The presence of Au dispersion on SrTiO_3_ caused chemo-mechanical strain, which resulted in oxygen vacancy at hetero-interfaces. With increased Au concentration, the generated chemo-mechanical strain changes the electronic band structure, resulting in an increase in the lowest unoccupied molecular orbital level. The strain effects presented may help to improve charge separation efficiency and adsorption site in water splitting. As a result, the photocatalytic activity was greatly boosted. All these studies suggest that interfacial defects due to lattice mismatch can improve photocatalytic water splitting performance by adjusting electronic structure, enhancing charge separation efficiency, and improving adsorption sites.

**Figure 16.**
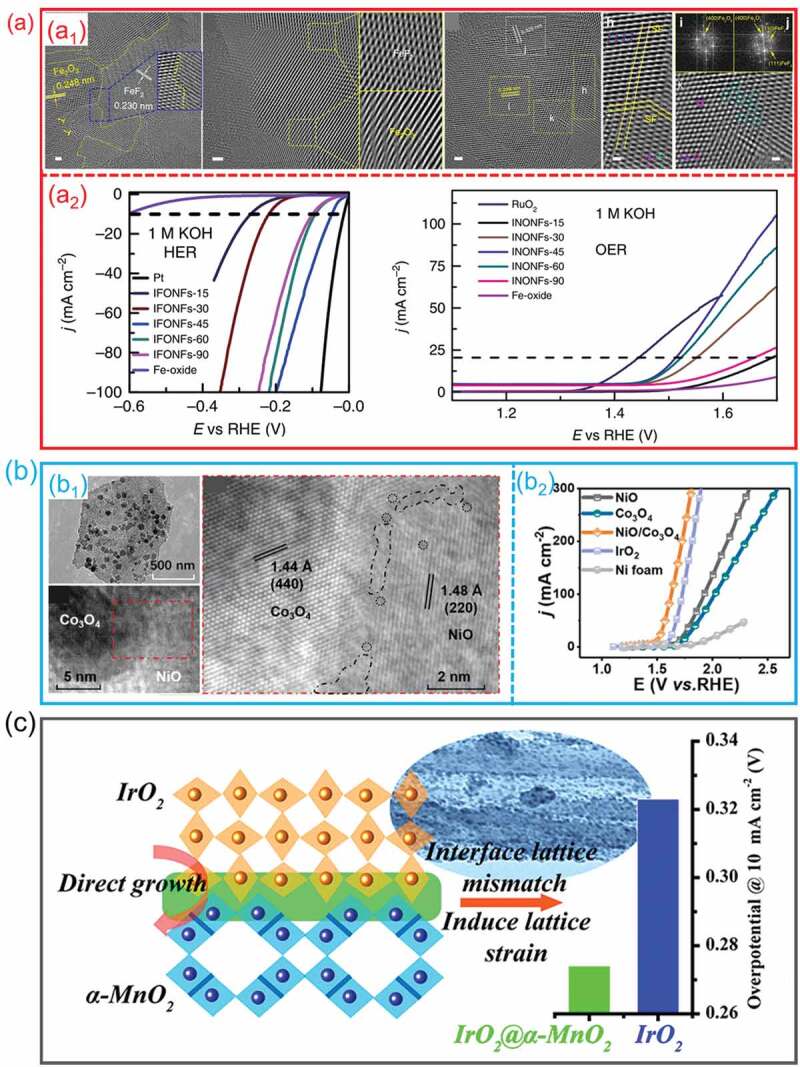
**The defect effect of hetero-interfaces for water splitting. (a) FeF**_2_**/Fe**_2_**O**_3_ **hetero-interfaces**: (a1) HRTEM images; (a2) HER and OER performance in 1 M KOH solution. (Reproduced with permission from [92], Copyright 2018, Nature) **(b) NiO/Co**_3_**O**_4_ **hetero-interfaces**: (b1) the TEM and HRTEM images; (b2) OER performance in 1 M KOH solution. (Reproduced with permission from [65], Copyright 2020, American Chemical Society) **(c)** Schematic illustration of the catalytic mechanism of IrO_2_/α-MnO_2_ hetero-interface with lattice strain defects. (Reproduced with permission from [131], Copyright 2017, American Chemical Society).

In electrocatalytic water splitting field, the defects that exist on hetero-interfaces have a significant impact on the chemical properties and electronic structures of the materials, and they have the potential to significantly boost the electrocatalytic efficacy of the materials when used to split water. For example, Fan et al. fabricated a FeF_2_/Fe_2_O_3_ hetero-interface by anodization/fluorination process [92]. The HRTEM images (Figure 16a_1_) show that the high-electrical-conductivity FeF_2_/Fe_2_O_3_ hetero-interface includes embedded disorder phases in the crystalline lattices, and it also features various distributed defects, such as interphase boundaries, stacking faults, oxygen vacancies, and dislocations on the surfaces and contact. This FeF_2_/Fe_2_O_3_ hetero-interface presents a remarkable electrocatalytic water splitting activity (Figure 16a_2_). Both empirical research and theoretical computations point to the fact that the surface and edge defects play a substantial role in the high performance of FeF_2_/Fe_2_O_3_ hetero-interface.

Zhang et al. demonstrated that the NiO/Co_3_O_4_ hetero-interfaces with oxygen vacancies at interface had an advanced OER performance [65]. TEM and HRTEM images show that that the abundant low-coordination atoms formed in NiO/Co_3_O_4_ hetero-interfaces led to an abundance of hetero-interfaces defects and increased oxygen vacancies at the interfaces (Figure 16b_1_). As a results, the NiO/Co_3_O_4_ hetero-interfaces presented a reduced overpotential and a shrinking Tafel slope (Figure 16b_2_). Theoretical calculations demonstrated that the *d* electrons were effectively modulated. Furthermore, the decrease of the unfavorable strong adsorption to oxo intermediates throughout the OER process was further confirmed by the observation that the d-band centers of Co at the interface in NiO/Co_3_O_4_ were distant from the Fermi level. Besides, various interfacial vacancies have been constructed, such as metal vacancies on LaCoO_3_/Co_3_O_4_ hetero-interface [32] and FeS_2_/CoS_2_ hetero-interface [132], oxygen vacancies on Ni_2_P/WO_2.83_ hetero-interface [133] and NiCo_2_O_4_/NiCoP hetero-interface [134], and nitrogen vacancy on *N*-NiMoO_4_/NiS_2_ hetero-interface [135], to improve the electrocatalytic water splitting property of electrocatalysts. In addition, Sun et al. demonstrated that introducing a lattice strain in IrO_2_/MnO_2_ hetero-interfaces due to interface lattice mismatch can improve the OER catalytic activity of IrO_2_ [131]. As shown in Figure 16(c), the lattice strain is created by growing IrO_2_ nanoparticles directly on a particularly exposed surface of α-MnO_2_ nanorods. They found the lattice mismatch in the underlying interface induces the formation of lattice strain in IrO_2_, which is the origins of performance enhancement rather than the charge transfer between the materials. Similarly, Zhu et al. also reported a MoS_2_/MoSe_2_ heterostructure with a precisely controlled the spatial scale of the hetero-interface region through a strain-driven synthesis method [95]. On the grain boundaries, an ultra-long nano-channel was formed, and it has been shown that this nano-channel may produce spontaneous strain at grain boundaries, which can then be used for the purpose of bandgap alteration as well as the efficient increase of catalytic performance in hydrogen generation. Liu et al. prepared a Ni_2_P/Co_2_P hetero-interface electrocatalyst with abundant stacking faults [94]. The stacking faults induce a tensile strain near the interfaces. This Ni_2_P/Co_2_P hetero-interface was employed as catalyst for H_2_O splitting and achieved an improved performance. They found that the tensile strain near Ni_2_P/Co_2_P hetero-interface can induce the d-band center shift and reduce the bandwidth to enhance the adsorption of reactants, leading to an enhancement electrocatalytic performance.

### 3.6 Synergic effect

**Figure 17.**
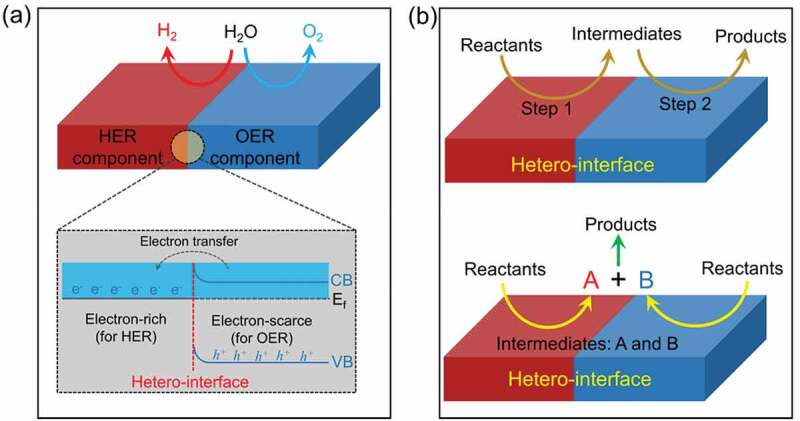
Illustration of the synergic effect on hetero-interfaces. (a) the hetero-interface synergistically facilitates the OER and HER processes. (b) the hetero-interface synergistically facilitates the reaction steps.

One of the properties of heterostructures that distinguishes them from single component materials is the synergistic effect induced by the presence of heterogeneous interfaces and multicomponent materials. The synergistic effect of hetero-interface has more applications in electrocatalytic water splitting, but is relatively rare in photocatalytic water splitting. Here, we mainly consider two types of synergic effect according to the geometric construction of hetero-interface, as shown in Figure 17. One involves synergistic facilitation of the OER and HER processes by compounding OER and HER components to create a hetero-interface (Figure 17(a)). In the other, the reaction steps are synergistically facilitated by establishing a hetero-interface (Figure 17(b)).

**Figure 18.**
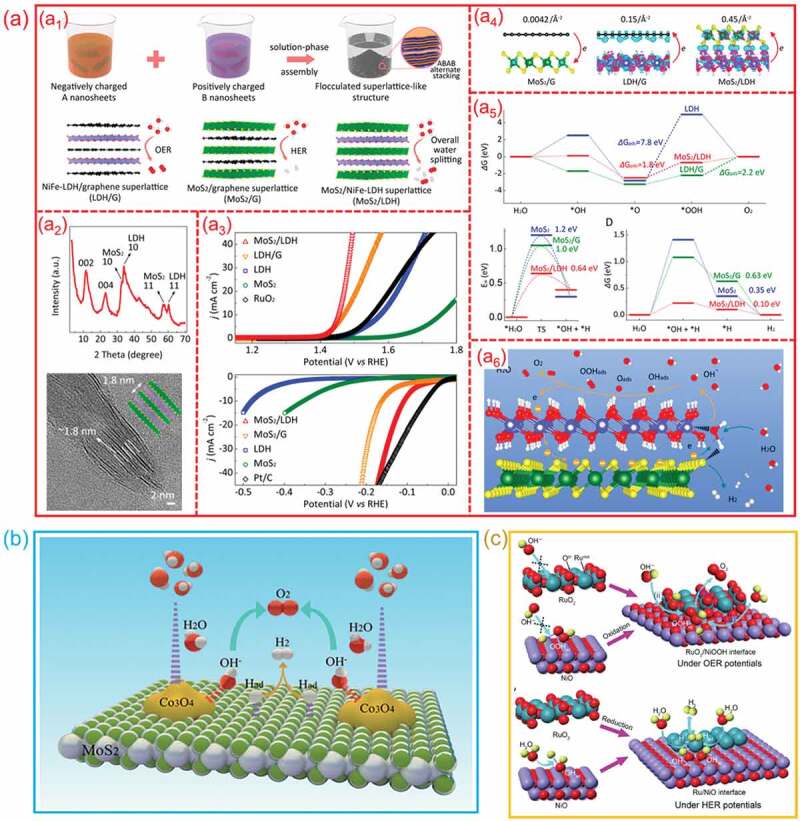
**The synergic effect of hetero-interfaces for overall water splitting. (a) MoS**_2_**/LDH hetero-interfaces**: (a1) Schematic of design and synthesis; (a2) the XRD pattern and HRTEM image; (a3) OER and HER performance; (a4) the distribution of differential charge density; (a5) Free energy diagrams of OER and HER; (a6) the possible mechanism of electrocatalytic water splitting process on the MoS_2_/LDH hetero-interface. (Reproduced with permission from [100], Copyright 2019, American Chemical Society) **(b) Co**_3_**O**_4_**/MoS**_2_ **hetero-interfaces**: Schematic of the HER and OER process at hetero-interfaces; (Reproduced with permission from [136], Copyright 2018, Royal Society of Chemistry) **(c) RuO**_2_**/NiO hetero-interfaces**: the potential-induced interfacial synergy between RuO_2_ and NiO for overall water splitting. (Reproduced with permission from [137], Copyright 2018, Wiley).

Generally, the catalysts with high activity for HER often exhibit a low activity for OER and *vice versa*. To take use of the benefits of both HER and OER electrocatalysts, one common and successful technique is to design a hetero-interface that compatibly integrates HER- and OER-active components. For instance, Xiong et al. constructed a series of 2D hetero-interfaces through electrostatic self-assembly method [100], including MoS_2_/NiFe-layered double hydroxide (MoS_2_/NiFe-LDH), MoS_2_/graphene (MoS_2_/G), and NiFe-LDH/G hetero-interfaces (Figure 18a_1_). The X-ray diffraction (XRD) and TEM results show that the existence of hetero-interfaces is in the form of superlattices (Figure 18a_2_). In alkaline electrolyte, the MoS_2_/NiFe-LDH hetero-interface shows a better OER and HER performance than that of MoS_2_/G and NiFe-LDH/G hetero-interfaces (Figure 18a_3_). The density functional theory (DFT) calculations show that the charge distributions on these hetero-interfaces is in the form of electron buildup at the interface. And the electron transport from one layer to the next. As shown in Figure 18a_4_, for MoS_2_/LDH hetero-interface, the electron transfer number from NiFe-LDH to MoS_2_ is 0.45 Å^−2^, which is much higher than that of LDH/G and MoS_2_/G hetero-interfaces. Further DFT findings indicate that firmly connecting NiFe-LDH with graphene or MoS_2_ significantly increases its OER activity, and the MoS_2_/LDH hetero-interface presents the most favorable reaction pathway for OER. For HER, the NiFe-LDH promotes the water dissociation process and favors the adsorption of *H intermediates on MoS_2_, thereby improving the HER performance (Figure 18a_5_). The synergistic effects at the MoS_2_/LDH interfaces result in a favorable energy barrier for the first water dissociation phase, facilitating H_2_ production. Notably, the considerable electron transfer from LDH to MoS_2_ finally improves both the HER and the OER processes on the MoS_2_ and LDH sides (Figure 18a_6_). Benefiting from the synergy between MoS_2_ and LDH, the MoS_2_/LDH hetero-interface realizes a remarkable overall water splitting in alkaline solution. Liu et al. constructed a mutually beneficial Co_3_O_4_/MoS_2_ hetero-structure which exhibit excellent property for overall water splitting performance [136]. As shown in Figure 18(b), by enhancing the sluggish kinetics, the Co_3_O_4_/MoS_2_ hetero-interface can effectively improve both HER and OER performance. For alkaline HER process, this hetero-interfaces can synergistically promote water dissociation and optimize the adsorption/desorption of H on MoS_2_ surface. Moreover, for OER process, this hetero-interface can enhance the adsorption of oxygen intermediates on Co_3_O_4_ surface. As a result, the Co_3_O_4_/MoS_2_ hetero-interface showed an outstanding overall water splitting activity in alkaline solution. In order to improve the overall water splitting property of RuO_2_, Liu et al. devised an efficient RuO_2_/NiO hetero-interface catalyst [137]. The potential-induced interfacial synergy between RuO_2_ and NiO is illustrated in Figure 18(c). Under the OER potentials, the discharge species OH^−^ is easily adsorbed on the hetero-interfaces to provide *OOH intermediates rather than on individual NiO or RuO_2_. The *OOH intermediates then go through an oxidation process to generate O_2_. For the HER part, the RuO_2_ was reduced to metallic Ru owing to the negative potentials. The H_2_O molecules were first dissociated on the NiO to provide *H for nearby Ru. Then, the combination of adjacent *H on Ru was achieved to produce H_2_. These results indicate that RuO_2_/NiO hetero-interface can cooperatively enhance the water decomposition performance.

**Figure 19.**
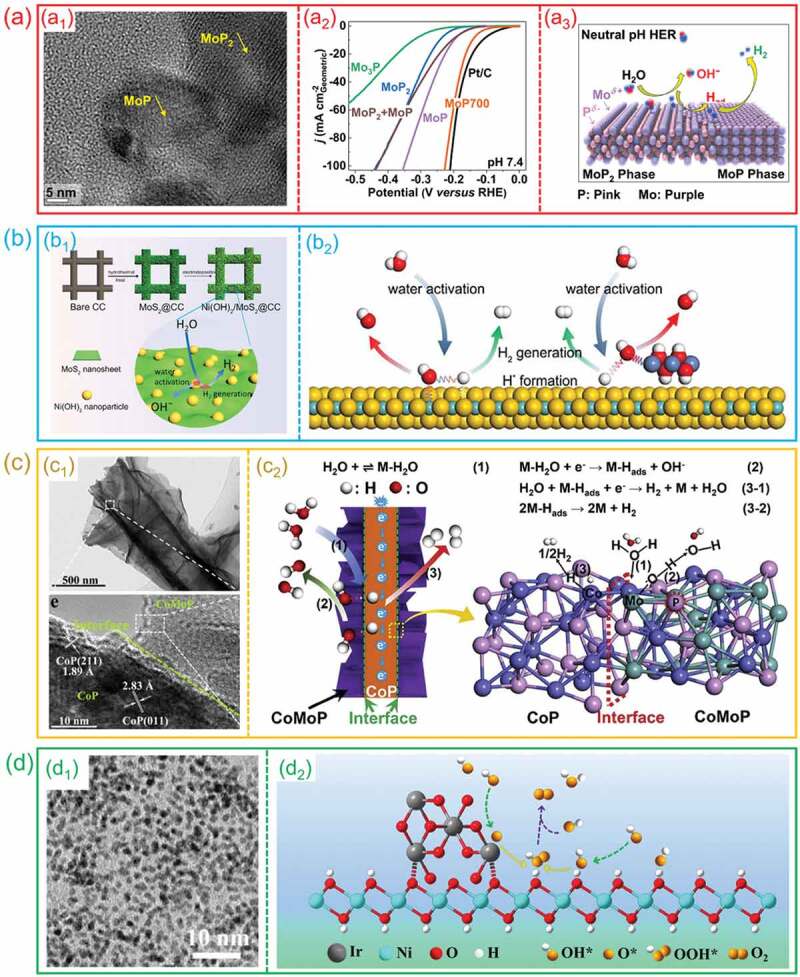
**The synergic effect of hetero-interfaces for HER and OER. (a) MoP/MoP**_2_ **hetero-interfaces for HER**: (a1) HRTEM image; (a2) the HER performance in neutral solution; (a3) the probable electrocatalytic mechanism on hetero-interfaces. (Reproduced with permission from [138], Copyright 2019, American Chemical Society) **(b) Ni(OH)**_2_**/MoS**_2_ **hetero-interfaces for HER**: (b1) the synthesis process of Ni(OH)_2_/MoS_2_ hetero-interface; (b2) the H_2_O activation, *H formation and H_2_ generation processes on hetero-interface. (Reproduced with permission from [64], Copyright 2017, Elsevier) **(c) CoP/comop hetero-interfaces for HER**: (c1) the representative TEM and HRTEM images; (c2) the synergistic enhancement effect of CoP/comop hetero-interfaces for HER. (Reproduced with permission from [97], Copyright 2020, Elsevier) **(d) Ir/Ni(OH)**_2_ **hetero-interfaces for OER**. (d1) the representative TEM image; (d2) the proposed OER process on Ir/Ni(OH)_2_ hetero-interface. Reproduced with permission from [139], Copyright 2020, Wiley.

Due to the lack of protons (H^+^), the HER process in neutral alkaline solutions begins with the adsorption and dissociation of H_2_O molecules as well as involves the recombination of *H. Thus, excellent adsorption performance for H_2_O molecules and desorption performance for *H are necessary factors for the efficient hydrogen evolution in neutral or alkaline electrolyte [53,72]. Xie et al. synthesized a “dual-active site” catalyst (MoP_2_/MoP) with high HER performance in neutral electrolyte [138]. As shown in Figure 19a_1_, the HRTEM results displayed that the coexistence of MoP_2_ and MoP in nanoscale and a well-defined hetero-interface was formed. In neutral electrolyte, the MoP_2_/MoP hetero-interface exhibits a lower overpotential, the HER performance of it is superior than that of single phosphides and very close to the commercial catalysts (Figure 19a_2_). Through DFT calculations, they found that, the Mo-MoP_2_ surfaces have enhanced H_2_O chemisorption property and the Mo−MoP surfaces have preferred H adsorption property, indicating that the synergistic effect of MoP_2_/MoP hetero-interface enhanced the performance of neutral HER (Figure 19a_3_). As shown in Figure 19b_1_, Zhang et al. constructed a highly efficient Ni(OH)_2_/MoS_2_ heterostructure alkaline HER electrocatalyst via a facile approach [64]. The DFT results demonstrate that the hydroxyl adsorption process easily occurs on the Ni(OH)_2_ and the produced hydrogen intermediates were adsorbed on the MoS_2_ active sites to generate H_2_ (Figure 19b_2_). Moreover, Huang et al. reported a highly efficient CoP/CoMoP catalyst with hetero-structures for HER [97]. As displayed in Figure 19c_1_, the corresponding TEM and HRTEM images of CoP/CoMoP showed that the CoMoP nanosheets is tightly grown on surface of the CoP nanowires and the hetero-interfaces between the crystalline CoP and the CoMoP is clear. The mechanism study reveals that CoMoP promotes the dissociation of water, while CoP facilitates the hydrogen adsorption. Benefiting from the synergy between CoP and CoMoP, CoP/CoMoP hetero-interfaces exhibited an excellent HER performance in alkaline solution (Figure 19c_2_).

In addition, synergistic effect also contributes to the OER process. As a typical example, Zhao et al. found that Ir/Ni(OH)_2_ hetero-interfaces can break the restrictive scaling relation in OER through the synergistic effect [139]. The TEM image shows that the Ir nanoparticles were uniformly distributed on the Ni(OH)_2_ nanosheets (Figure 19d_1_). The Ir/Ni(OH)_2_ hetero-interface exhibits a low overpotential for OER, which is superior than that of pristine Ni(OH)_2_ and Ir. As shown in Figure 19d_2_, the mechanism investigation demonstrates that OER intermediate are adsorbed on different active substances respectively. The interaction of the two intermediates at the heterostructure interface promotes the synthesis of OOH*, breaking the scaling relation and resulting in significantly accelerated OER kinetics.

## 4. Conclusions and perspectives

Designing hetero-interface has been acknowledged as a promising strategy for exploiting high-efficiency catalysts for photocatalytic and electrocatalytic water splitting reaction. In this paper, we have systematically reviewed the fundamental understanding and recent development of nanoscale hetero-interfaces for water splitting in the fields of photocatalysis and electrocatalysis. Hetero-interfaces have the characteristics of band alignment, space charge separation, charge/electron/mass transfer, support effect, defect effect, and synergistic effect, which exhibit modified band structure, efficient charge separation, lower reaction energy barriers, faster charge/electron/mass transfer, more active sites, higher conductivity, and higher stability during water splitting process. By rationally designing the hetero-interfaces of catalysts, the water splitting process can be effectively promoted for the rapid production of green hydrogen energy.

Although considerable progress of hetero-interfaces for electrocatalysis and photocatalysis have been achieved, there are still some challenges should be considered. (1) Precisely controllable the fabricating hetero-interface is difficult. There are various existing hetero-interface synthesis techniques, but there is no universal synthesis method to prepare hetero-interfaces with designated configurations. Moreover, most of the existing synthesis methods are difficult to precisely control the configuration of the hetero-interface, especially the realization of precise control at the atomic level. Therefore, it is necessary to develop a universal and easy-to-control interface synthesis technology to precisely synthesize and control the configuration of the hetero-interface. (2) Ascertaining the structure of hetero-interface is difficult. The structure of hetero-interfaces will be changed during catalytic process. Although a large number of characterization techniques have been used to observe the structure of hetero-interfaces, these characterization techniques are basically ex-situ characterization and it is difficult to observe the interface changes during the catalytic process in real time. The mechanism and process of interface structure change during catalysis are still unclear. An in-depth understanding of the change mechanism of the interface structure and the exploration of the reasons for the changes in activity and stability during the catalytic process can provide guidance for the design of more efficient and stable interface structure catalysts. Therefore, it is necessary to develop new in-situ characterization techniques to study the dynamic evolution of the interface during the catalytic process. (3) Understanding the catalytic mechanism on hetero-interface is difficult. Due to the complexity of the interface and the instability of the structure, it is difficult to study the catalytic reaction mechanism at the interface. This requires us to synthesize a well-defined and stable interface, and to develop in-situ techniques to observe the catalytic process on the interface, so as to establish a reliable structure-activity relationship.
